# Hydroclimate response of spring ecosystems to a two-stage Younger Dryas event in western North America

**DOI:** 10.1038/s41598-022-11377-4

**Published:** 2022-05-05

**Authors:** Jeffrey S. Pigati, Kathleen B. Springer

**Affiliations:** grid.2865.9 0000000121546924 U.S. Geological Survey, Denver Federal Center, MS 980, Box 25046, Denver, CO 80225 USA

**Keywords:** Climate sciences, Palaeoclimate

## Abstract

The Younger Dryas (YD) climate event is the preeminent example of abrupt climate change in the recent geologic past. Climate conditions during the YD were spatially complex, and high-resolution sediment cores in the North Atlantic, western Europe, and East Asia have revealed it unfolded in two distinct stages, including an initial stable climatic period between ~ 12.9 and 12.2 ka associated with a weakened Atlantic Meridional Overturning Circulation (AMOC) and a second phase characterized by variable conditions until 11.7 ka as the AMOC recovered. Decades of investigations into the climate of western North America during the YD have failed to identify this stepped phenomenon. Here we present hydroclimate data from paleospring deposits in Death Valley National Park (California, USA) that demonstrate unequivocal evidence of two-stage partitioning within the YD event. High groundwater levels supported persistent and long-lived spring ecosystems between ~ 13.0 and 12.2 ka, which were immediately replaced by alternating wet and dry environments until ~ 11.8 ka. These results establish the mid-YD climate transition extended into western North America at approximately the same time it was recorded by hydrologic systems elsewhere in the Northern Hemisphere and show that even short-lived changes in the AMOC can have profound consequences for ecosystems worldwide.

## Introduction

The Atlantic Meridional Overturning Circulation (AMOC) is one of the world’s key ocean circulation systems, transporting warm water from the tropics to the polar North Atlantic and cooler water southward along the ocean floor^1^. This transport of heat poleward is a fundamental driver of the global climate system, so when the AMOC is interrupted, there are profound consequences for climate and ecosystems around the world^2^. Recent observations and empirical data indicate a gradual weakening of the AMOC has occurred over the past century, and the system may be approaching a point close to a critical transition^3^. To determine the potential impacts and provide context for the cascading effects of reaching such a tipping point^4^, it is imperative to examine past episodes of abrupt climate change brought about by perturbations in the AMOC.

The Younger Dryas (YD) climate event was the final major climatic fluctuation of the last glacial period, spanning from ~ 12.9 to 11.7 ka (ka = thousands of years before present; 0 ka = 1950 CE)^5^, and is attributed to abrupt weakening of the AMOC^6,7^. The YD event featured climate conditions that were spatially complex, including pronounced cooling at high latitudes in the Northern Hemisphere, particularly in the North Atlantic region, lesser cooling in the tropics and subtropics, and slight warming in the Southern Hemisphere^8^, and therefore represents an ideal natural laboratory for studying the effects of rapid shifts in the global climate system.

**Figure 1:**
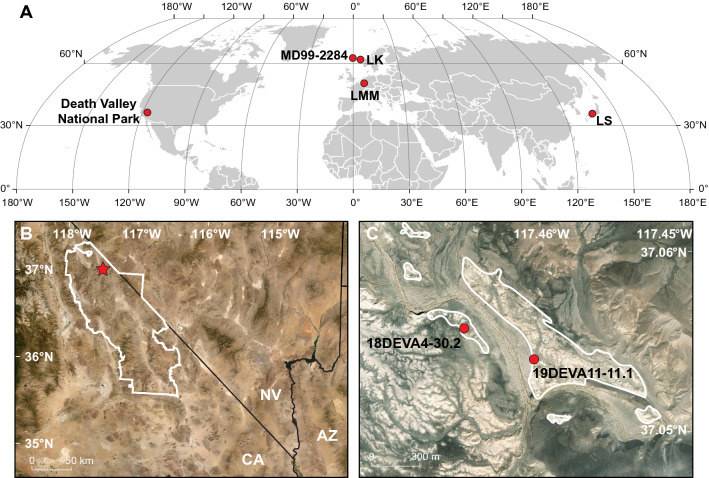
(**a**) Locations of sites where unequivocal evidence of a two-stage Younger Dryas (YD) climate event has been documented. Death Valley National Park; MD99-2284 = marine core in the Faeroe-Shetland passage off the coast of Norway^16^; LK = Lake Kråkenes, Norway^16^; LMM = Lake Meerfelder Maar, Germany^14^; LS = Lake Suigetsu, Japan^20^. (**b**) Location of Death Valley National Park (white line) in the southwestern U.S. AZ = Arizona, CA = California, NV = Nevada. Red star shows the location of the current study. (**c**) Locations of key stratigraphic sections, 19DEVA11-11.1 (11S, 459157 m E, 4100907 m N) and 18DEVA4-30.2 (11S, 458705 m E, 4101050 m N) (red circles), and the spatial extent of YD-aged spring deposits in the basin (white lines).

The impacts of the YD on ecosystems worldwide have been investigated for decades, with particular emphasis placed on environmental changes that occurred either at the onset or the termination of the event e.g.^9–11^. However, climate variability *within* the YD has received far less scrutiny. Some high-resolution lacustrine and marine records from the North Atlantic, western Europe, and East Asia (Fig. 1a) have documented intra-YD variations in hydroclimate that are linked to shifts in the mean states of ocean circulation patterns and associated atmospheric regimes brought about by changes in the AMOC^12–20^. These studies determined the YD event consisted of two distinct stages, including an early stable period between ~ 12.9 and 12.2 ka related to the weakening of the AMOC and in which key climate parameters show little variation, followed by a transition into an unstable phase between ~ 12.2 and 11.7 ka characterized by highly variable conditions as the AMOC began to recover.

Whether the two-stage nature of the YD climate event extended throughout the entire Northern Hemisphere is unclear. In western North America, for example, decades of investigations have focused on various aspects of the YD without clearly documenting this stepped phenomenon. We reviewed more than 200 studies from western U.S., Canada, and Mexico (SI1) and found that although a few recognize potential evidence of climatic variations within the YD, interpretations of these records can be contradictory, proxies within individual studies often do not maintain internal consistency, and/or chronologic control is insufficient to determine the precise timing of a mid-YD transition (SI2). Climate models used to reconstruct late Pleistocene hydroclimate conditions in western North America have also failed to reconstruct intra-YD partitioning^21^.

In the southwestern U.S., lacustrine and nearshore marine records, speleothems, pollen sequences, packrat middens, and dunes have established that, with few exceptions^22,23^, climate conditions during the YD were overwhelmingly cool and/or wet^24,25^. Throughout the Mojave Desert, geologic deposits associated with desert spring ecosystems show that water tables were high during the YD, and the transitions into and out of these wet conditions were synchronous with the beginning and end of the YD, respectively^26–28^, demonstrating their dynamic response to abrupt climate change^29,30^. Records from spring ecosystems have also captured evidence of fluctuating groundwater levels within the YD^31^ but have not yet identified clear evidence of a two-stage climate event.

This study presents new hydroclimate data from paleospring deposits in Death Valley that exhibit the rare combination of variables required to capture the two-stage nature of the YD event, including exceptionally high sedimentation rates, an ecosystem that is extremely sensitive to climatic fluctuations, and datable materials at key stratigraphic intervals. Our results show unequivocally that climate partitioning *within* the YD extended into western North America and the timing of the mid-YD transition was similar to the transition recorded by hydrologic systems elsewhere in the Northern Hemisphere.

## Death Valley paleospring deposits

In the northern part of Death Valley National Park, California, a thick (~ 20 m) package of light-colored, fine-grained sediments exhibiting badland topography occupies several square kilometers of a structural basin (Fig. 1b). These deposits represent spring ecosystems that formed in the valley bottom as groundwater levels responded to changing hydroclimate conditions during the late Quaternary. The sedimentary sequence includes carbonate-rich sediments that represent discrete spring hydrologic regimes (marshes, wet meadows, spring-fed pools and streams) and bear a remarkable sedimentologic and stratigraphic resemblance to paleospring deposits elsewhere in the Mojave Desert^28–30^.

Inset within this larger sequence, the youngest part of the record is especially well-preserved along the valley axis, in the topographically lowest part of the basin, where a distinctive lithologic sequence covers nearly one square kilometer (Fig. 1c). This ~ 4 m thick unit occurs in outcrops that can be traced laterally without interruption for hundreds of meters. We chose two stratigraphic sections that best represented the characteristics of the deposits to describe, measure, and sample. The resulting paleohydrologic interpretations are based on observation of modern spring ecosystem analogs and were applied to this geologic record following Springer et al.^30^.

**Figure 2:**
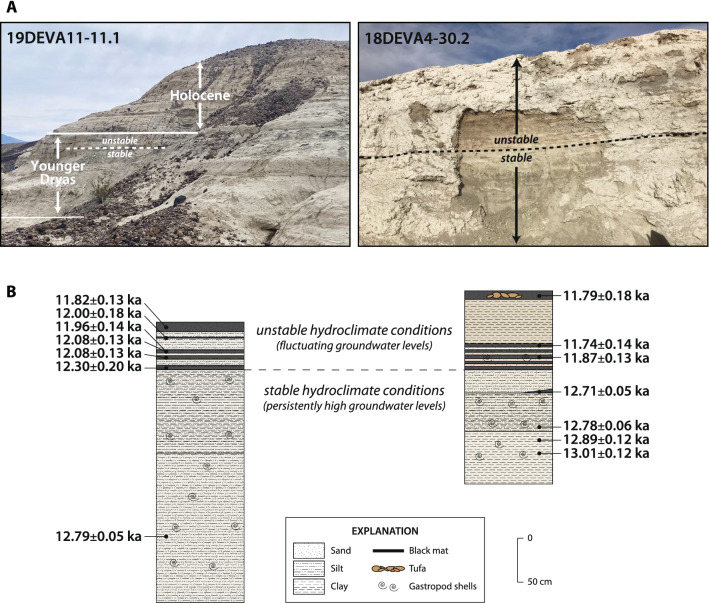
(**a**) Annotated photographs and (**b**) stratigraphic sections of paleospring deposits dating to the Younger Dryas (YD) climate event. Dashed lines represent the stratigraphic transition from high groundwater levels and stable hydroclimate conditions to lower and variable water table levels associated with a drier, unstable hydroclimate within the YD. Details of the dating methods and results, as well as additional sedimentologic data, are included in SI3 and 4, respectively. Photographs courtesy of the authors.

The spring deposits in this part of the basin exhibit two clear-cut modes of sedimentation (Fig. 2). The lower 2–3 m of sediments consist of massive, olive-green silt and clay with abundant aquatic gastropods (*Gyraulus* sp.) and bivalves (*Pisidium* sp.) as well as numerous terrestrial gastropods (Succineidae, Pupillidae). The sedimentologic and faunal components of this unit are collectively indicative of a marshy environment with reducing conditions. In sharp contact with this lower sequence is an upper ~ 1 m of sediment exhibiting numerous, thin (5–10 cm) black mats (organic-rich silt and clay) containing a few terrestrial gastropod shells (Succineidae) that are interbedded repeatedly with layers of massive, oxidized eolian sands lacking microfauna. These contrasting hydrologic settings represent alternating periods of groundwater discharge (black mats with gastropods) and aridity (eolian sands).

**Figure 3:**
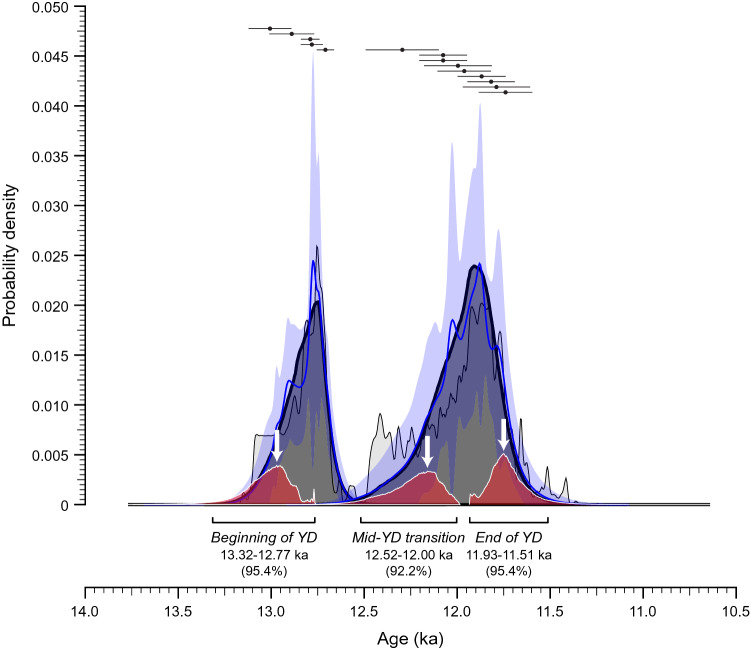
Kernel density estimate (KDE) modeling results for the calibrated ^14^C ages (filled dark circles with uncertainties presented at the 95% (2σ) confidence level). The KDE distribution is shown in dark gray outlined in black. The blue line and lighter blue band show the mean and ± 1σ for snapshots (n = 1000) of the KDE distribution, which give an indication of the significance of individual features. The Sum distribution is shown in light gray and the start and end of the Boundary distribution are in red. The white arrows show the maximum probability density for the beginning (~ 13.0 ka) and end (~ 11.8 ka) of the YD sedimentary sequence, as well as the transition between the two phases of sedimentation (~ 12.2 ka). Generated using the KDE_Model and Boundary functions in OxCal v.4.4.2 r:5^32^ and the IntCal20 calibration curve^40^; accessed online 02/25/2021.

We used radiocarbon dating of charcoal, organic matter, and terrestrial gastropod shells (Succineidae) to date the sedimentary sequences at both locations (SI3). We also applied the kernel density estimation (KDE) model (KDE_Model) function of OxCal4.4.2^32^ to determine the underlying distribution of the discrete data points, and the OxCal Boundary function to constrain the start and end dates of the subunits, as well as the timing of the change in sedimentation. The calibrated ages and modeling results reveal the entire depositional sequence spans from ~ 13.0 to 11.8 ka (Fig. 3), comparable to the timing of the YD climate event in the NGRIP ice core record^5^ when considering the uncertainties in both the ice core chronology (~ 100 years) and our ages (~ 100-200 years). Additionally, the results constrain the timing between the distinct lower and upper sedimentary phases to ~ 12.2 ka (Fig. 3).

The YD depositional sequence in Death Valley exhibits the highest sedimentation rate (~ 2.7 mm/year) observed in paleospring deposits anywhere in the southwestern U.S. This extraordinary record allowed us to examine the response of spring ecosystems there to subtle changes in climate at an unusually high temporal resolution and to evaluate hydroclimate conditions within the YD event at a level that has never been achieved before in geologic outcrops. The results of our study show that high groundwater levels in the basin supported persistent and long-lived spring ecosystems during the first part of the YD climate event, beginning at ~ 13.0 ka and continuing until 12.2 ka. This was followed by alternating wet and dry environments through the remainder of the event until ~ 11.8 ka associated with lower and variable water table levels and drier hydroclimate conditions.

## Climatic transition within the Younger Dryas across the northern hemisphere

**Figure 4:**
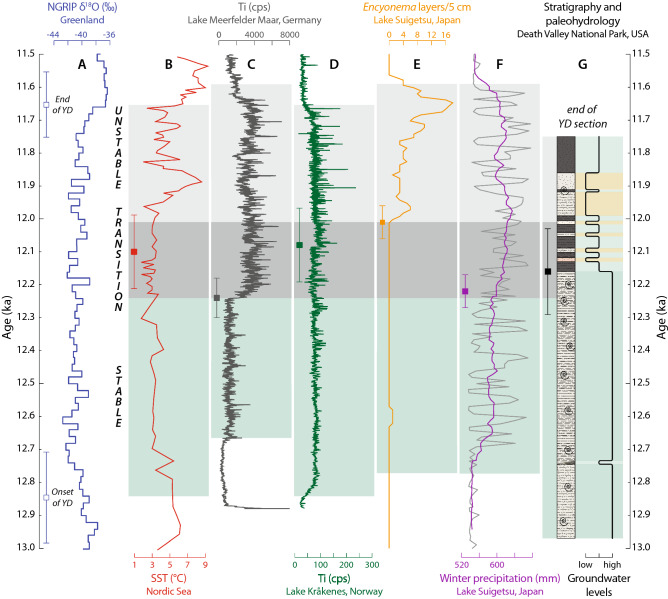
Paleospring record from Death Valley National Park, California (DEVA) compared to other proxy records that exhibit evidence of two-stage partitioning during the Younger Dryas (YD) climate event. Ages for all records are presented in ka (ka = thousands of calendar years before present; 0 ka = 1950 A.D.), and uncertainties are presented at the 95% (2σ) confidence level. Open squares in A show the timing of the onset and end of the YD in the NGRIP ice core record^5^. Filled squares in B-G show the timing of the transition between the two stages of the YD as defined in the original studies with the corresponding 2σ uncertainties. Dark green bands show the stable phase of the event for each record, the light gray bands denote unstable conditions, and the dark gray bands encompass the full temporal range of the transition between the two stages. (**a**) Oxygen isotope (δ^18^O) values from the NGRIP ice core using the GICC05 chronology^5^. (**b**) Sea-surface temperatures (SSTs) reconstructed from planktonic foraminiferal assemblages in marine core MD99-2284 off the coast of Norway^16^. (**c**) Micro–X-ray fluorescence (XRF) measurements of Ti (in counts per second; cps) in sediments from Lake Meerfelder Maar, Germany as a proxy for detrital flux in spring snowmelt^14,18^. (**d**) Ti in sediments from Lake Kråkenes, Norway as a proxy for mass turnover rates of the Kråkenes glacier^16,18^. (**e**) Layers rich in the diatom *Encyonema* in sediments from Lake Suigetsu, Japan as a proxy for wind strength and turbulence^20^. (**f**) Pollen-based reconstruction of winter precipitation at Lake Suigetsu^20^. (**g**) Composited stratigraphy (left) and inferred groundwater levels (solid black line, right) of the DEVA paleospring deposits (this study). On the far right, dark green bands represent stable, long-lived spring ecosystems; light green bands denote short-lived springs; tan bands mark periods of aridity.

The Death Valley spring deposits demonstrate the two-stage nature of the YD event extended into western North America and show the timing of the transition between the two stages of the YD event was similar to the timing of the mid-YD transition elsewhere in the Northern Hemisphere (Fig. 4). In the North Atlantic, an increase in freshwater base discharge that weakened the AMOC and initiated the YD event at ~ 12.9 ka^7,33–36^ was followed by a period of several hundred years during which the AMOC remained in a weakened state. The AMOC then began to recover between ~ 12.4 to 12.3 and 12.0 ka as freshwater discharge decreased^37^. In response, cool and relatively constant sea-surface temperatures (SSTs) in the Nordic Sea that prevailed during the early part of the YD yielded to warmer and more variable SSTs beginning at 12.10 ± 0.11 ka (Fig. 4b)^16^. In western Europe, reorganizations of atmospheric circulation regimes associated with recovery of the AMOC caused an increase in spring snowmelt at 12.24 ± 0.06 ka at Lake Meerfelder Maar, Germany (Fig. 4c)^14^ and increased mass turnover rates of nearby glaciers at 12.08 ± 0.11 ka near Lake Kråkenes, Norway (Fig. 4d)^16^. In East Asia, an increase in winter precipitation within the YD inferred from pollen at Lake Suigetsu, Japan is dated to 12.22 ± 0.05 ka, and lake turbulence based on diatom assemblages increased abruptly at 12.01 ± 0.05 ka (Fig. 4e,f)^20^. Finally, in the western part of North America, the Death Valley spring deposits show that water tables were high and stable until 12.16 ± 0.13 ka before they dropped and fluctuated for the remainder of the event (Fig. 4g).

The impact that the mid-YD recovery of the AMOC had on these different hydroclimate systems varied by geographic location, as the climate characteristics (cold versus warm, wet versus dry) in the North Atlantic, western Europe, and western North America were reversed in East Asia. Importantly, the mid-YD transition did not occur simultaneously across the Northern Hemisphere as it was time-transgressive in western Europe^18^ and was recorded at different times by various proxies at Lake Suigetsu^20^. In addition, although the mid-YD transition appears to have occurred relatively quickly in all of these studies, on the order of years to decades, other archives suggest the transition took place over several centuries^19^. Despite these complexities, the disparate records collectively demonstrate that the transition from stable to unstable hydroclimate conditions within the YD event occurred between ~ 12.2 and 12.0 ka.

Propagation of the mid-YD transition across the Northern Hemisphere was faster than can be delineated in these proxy records, implying transmission occurred via atmospheric teleconnections^38^. Following Schlolaut et al.^20^, we hypothesize that significant reorganizations of hydroclimate conditions caused a deepening of the Aleutian Low and a northward shift in the westerlies as the AMOC began to reestablish itself between ~ 12.4 to 12.3 and 12.0 ka. This forced repositioning of temperature and precipitation zones across Europe, Asia, and North America, and resulted in the hydroclimate changes in the southwestern U.S. recorded by the Death Valley spring deposits.

The specific amount of annual precipitation in this region of the Mojave Desert during either stage of the YD event is unknown, as is the magnitude of the decrease in precipitation at ~ 12.2 ka that marks the boundary between the two stages. Recent transient climate simulations do not show a difference in climatic conditions across the two stages of the YD event, and in fact fail to reconstruct wet conditions that prevailed in this region during the event as a whole^21^. The hydrologic record presented here documents high groundwater levels persisted in Death Valley during the entirety of the YD event and provides robust evidence of the two-stage nature of the event itself, setting a new standard for climate modeling and other studies aimed at investigating hydroclimate conditions in western North America during the late Pleistocene.

The Death Valley record also demonstrates that teleconnections between the North Atlantic and southwestern U.S. are strong enough that even short-lived changes in the AMOC can dramatically affect ecosystems in the American Southwest. New investigations and re-examination of existing high-resolution geologic records are needed to define the full breadth and spatial extent of intra-YD hydroclimate variations in western North America and elsewhere, thereby aiding our understanding of the potential impacts of future changes in the AMOC. Such studies also create the temporal link to extant desert springs and wetlands, which are keystone ecosystems in arid environments worldwide, and provide critical baseline data for their effective stewardship^39^.

## Supplementary Information

Supplementary Information.
