# Supplementary material for: Hydroclimate response of spring ecosystems to a two-stage Younger Dryas event in western North America
Source: Sci Rep. 2022 May 5;12:7323. doi: 10.1038/s41598-022-11377-4 (PMC9072542; doi:10.1038/s41598-022-11377-4)
Supplement: Supplementary file 1 — Supplementary Information. [file 41598_2022_11377_MOESM1_ESM.pdf]

Pigati, J.S., Springer, K.B., Hydroclimate response of spring ecosystems to a two-stage Younger Dryas event in western North America. *Nature Scientific Reports*.

Captions for the Supplemental Information files*

SI 1. Citations for more than 200 articles reviewed as part of this study that explicitly recognize the Younger Dryas (YD) climate event in western North America. The citations are ordered by region, then alphabetically by lead author.

SI 2a. Of the articles listed in SI 1, only nine studies recognized hydroclimate variability within the YD event. Here we provide a summary of the site locations, proxies measured, and hydroclimate interpretations, as well as an assessment of whether the data represent unequivocal evidence of a two-stage YD event. The studies are ordered by state, then alphabetically by site.

SI 2b. Summary of site information, type of deposit, record type, age range and number of dates within the YD chronozone, and the proxies measured in each of the nine studies included in SI 2a.

SI 3a. Description of laboratory methods and age modeling used in this study.

SI 3b. Summary of sample information, radiocarbon ages, and calibrated ages for samples collected from the DEVA paleospring deposits.

SI 4. Sedimentologic data for sections 18DEVA4-30.2 (top) and 19DEVA11.11.1 (bottom).

*Downloadable files of the data presented in the Supplementary Information can be found at https://doi.org/10.5066/P9U5XSRY.

SI 1

Alaska

Ager, T. A., 2019, Late Quaternary vegetation development following deglaciation of northwestern Alexander Archipelago, Alaska: Frontiers in Earth Science, v. 7, doi: 10.3389/feart.2019.00104.

Ager, T. A., Carrara, P. E., Smith, J. L., Anne, V., and Johnson, J., 2010, Postglacial vegetation history of Mitkof Island, Alexander Archipelago, southeastern Alaska: Quaternary Research, v. 73, p. 259-268.

Ager, T. A., and Rosenbaum, J. G., 2007, Late glacial-Holocene pollen-based vegetation history from Pass Lake, Prince of Wales Island, southeastern Alaska: U.S. Geological Survey Professional Paper 1760-G, 19 p.

Badding, M. E., Briner, J. P., and Kaufman, D. S., 2013, ^{10}Be ages of late Pleistocene deglaciation and Neoglaciation in the north-central Brooks Range, Arctic Alaska: Journal of Quaternary Science, v. 28, no. 1, p. 95-102.

Bigelow, N., Begét, J., and Powers, R., 1990, Latest Pleistocene increase in wind intensity recorded in eolian sediments from central Alaska: Quaternary Research, v. 34, p. 160-168.

Bigelow, N. H., and Edwards, M. E., 2001, A 14,000 yr paleoenvironmental record from Windmill Lake, central Alaska: Lateglacial and Holocene vegetation in the Alaska range: Quaternary Science Reviews, v. 20, p. 203-215.

Bigelow, N. H., and Powers, W. R., 2001, Climate, vegetation, and archaeology 14,000-9000 cal yr B.P. in central Alaska: Arctic Anthropology, v. 38, no. 2, p. 171-195.

Briner, J. P., and Kaufman, D. S., 2008, Late Pleistocene mountain glaciation in Alaska: Key chronologies: Journal of Quaternary Science, v. 23, no. 6/7, p. 659–670.

Briner, J. P., Kaufman, D. S., Werner, A., Caffee, M., Levy, L., Manley, W. F., Kaplan, M. R., and Finkel, R. C., 2002, Glacier readvance during the late glacial (Younger Dryas?) in the Ahklun Mountains, southwestern Alaska: Geology, v. 30, no. 8, p. 679-682.

Brubaker, L. B., Anderson, P. M., and Hu, F. S., 2001, Vegetation ecotone dynamics in southwest Alaska during the Late Quaternary: Quaternary Science Reviews, v. 20, p. 175-188.

Engstrom, D. R., Hansen, B. C. S., and Wright, H. E., Jr., 1990, A possible Younger Dryas record in southeastern Alaska: Science, v. 250, p. 1383-1385.

Finkenbinder, M. S., Abbott, M. B., Finney, B. P., Stoner, J. S., and Dorfman, J. M., 2015, A multi-proxy reconstruction of environmental change spanning the last 37,000 years from Burial Lake, Arctic Alaska: Quaternary Science Reviews, v. 126, p. 227-241.

Hajdas, I., Bonani, G., Bodén, P., Peteet, D. M., and Mann, D. H., 1998, Cold reversal on Kodiak Island, Alaska, correlated with the European Younger Dryas by using variations of atmospheric ^{14}C content: Geology, v. 26, no. 11, p. 1047-1050.

Hansen, B. C. S., and Engstrom, D. R., 1996, Vegetation history of Pleasant Island, southeastern Alaska, since 13,000 yr B.P.: Quaternary Research, v. 46, p. 161-175.

Hu, F. S., Brubaker, L. B., and Anderson, P. M., 1995, Postglacial vegetation and climate change in the northern Bristol Bay region, southwestern Alaska: Quaternary Research, v. 43, p. 382-392.

Hu, F. S., Lee, B. Y., Kaufman, D. S., Yoneji, S., Nelson, D. M., and Henne, P. D., 2002, Response of tundra ecosystem in southwestern Alaska to Younger-Dryas climatic oscillation: Global Change Biology, v. 8, p. 1156-1163.

Hu, F. S., Nelson, D. M., Clarke, G. H., Rühland, K. M., Huang, Y., Kaufman, D. S., and Smol, J. P., 2006, Abrupt climatic events during the last glacial-interglacial transition in Alaska: Geophysical Research Letters, v. 33, p. L18708, doi: 18710.11029/12006GL027261.

Jones, M. C., Peteet, D. M., Kurdyla, D., and Guilderson, T., 2009, Climate and vegetation history from a 14,000-year peatland record, Kenai Peninsula, Alaska: Quaternary Research, v. 72, p. 207-217.

Kaufman, D. S., Anderson, R. S., Hub, F. S., Berg, E., and Werner, A., 2010, Evidence for a variable and wet Younger Dryas in southern Alaska: Quaternary Science Reviews, v. 29, p. 1445-1452.

Kokorowski, H., D,, Anderson, P. M., Mock, C. J., and Lozhkin, A. V., 2008, A re-evaluation and spatial analysis of evidence for a Younger Dryas climatic reversal in Beringia: Quaternary Science Reviews, v. 27, p. 1710-1722.

Kurek, J., Cwynar, L. C., Ager, T. A., Abbott, M. B., and Edwards, M. E., 2009, Late Quaternary paleoclimate of western Alaska inferred from fossil chironomids and its relation to vegetation histories: Quaternary Science Reviews, v. 28, p. 799-811.

Peteet, D. M., and Mann, D. H., 1994, Late-glacial vegetational, tephra, and climatic history of southwestern Kodiak Island, Alaska: Ecoscience, v. 1, no. 3, p. 255-267.

Wilcox, P. S., Fowell, S. J., and Baichtal, J. F., 2020, A mild Younger Dryas recorded in southeastern Alaska: Arctic, Antarctic, and Alpine Research, v. 50, p. 236-247.

Young, N. E., Briner, J. P., Schaefer, J., Zimmerman, S., and Finkel, R. C., 2019, Early Younger Dryas glacier culmination in southern Alaska: Implications for North Atlantic climate change during the last deglaciation: Geology, v. 47, p. 550-554.

Yu, Z., Walker, K. N., Evenson, E. B., and Hajdas, I., 2008, Lateglacial and early Holocene climate oscillations in the Matanuska Valley, south-central Alaska: Quaternary Science Reviews, v. 27, p. 148-161.

Cordilleran (USA and Canada)

Anderson, R. S., Allen, C. D., Toney, J. L., Jass, R. B., and Bair, A. N., 2008a, Holocene vegetation and fire regimes in subalpine and mixed conifer forests, southern Rocky Mountains, USA: International Journal of Wildland Fire, v. 17, p. 96-114.

Anderson, R. S., Jass, R. B., Toney, J. L., Allen, C. D., Cisneros-Dozal, L. M., Hess, M., Heikoop, J., and Fessenden, J., 2008b, Development of the mixed conifer forest in northern New Mexico and its relationship to Holocene environmental change: Quaternary Research, v. 69, p. 263-275.

Anderson, R. S., Soltow, H. R., and Jiménez-Moreno, G., 2018, Postglacial environmental change of a high-elevation forest, Sangre de Cristo Mountains of south central Colorado, in Starratt, S. W., and Rosen, M. R., eds., From Saline to Freshwater: The Diversity of Western Lakes in Space and Time: Geological Society of America Special Paper 536, p. 1-20, doi: 10.1130/2018.2536(1113).

Armour, J., Fawcett, P. J., and Geissman, J. W., 2002, 15 k.y. paleoclimatic and glacial record from northern New Mexico: Geology, v. 30, no. 8, p. 723-726.

Beierle, B. D., Smith, D. G., and Hills, L. V., 2003, Late Quaternary glacial and environmental history of the Burstall Pass area, Kananaskis Country, Alberta, Canada: Arctic, Antarctic, and Alpine Research, v. 35, no. 3, p. 391-398.

Benson, L., Madole, R., Kubik, P., and McDonald, R., 2007, Surface-exposure ages of Front Range moraines that may have formed during the Younger Dryas, 8.2 cal ka, and Little Ice Age events: Quaternary Science Reviews, v. 26, p. 1638-1649.

Birkel, S. D., Putnam, A. E., Denton, G. H., Koons, P. O., Fastook, J. L., Putnam, D. E., and Maasch, K. A., 2012, Climate inferences from a glaciological reconstruction of the late Pleistocene Wind River ice cap, Wind River Range, Wyoming: Arctic, Antarctic, and Alpine Research, v. 44, no. 3, p. 265-276.

Briles, C. E., Whitlock, C., and Meltzer, D. J., 2012, Last glacial–interglacial environments in the southern Rocky Mountains, USA and implications for Younger Dryas-age human occupation: Quaternary Research, v. 77, p. 96-103.

Corbett, L. R., and Munroe, J. S., 2010, Investigating the influence of hydrogeomorphic setting on the response of lake sedimentation to climatic changes in the Uinta Mountains, Utah, USA: Journal of Paleolimnology, v. 44, p. 311-325.

Dahms, D., Egli, M., Fabel, D., Harbor, J., Brandová, D., de Castro Portes, R., and Christl, M., 2018, Revised Quaternary glacial succession and post-LGM recession, southern Wind River Range, Wyoming, USA: Quaternary Science Reviews, v. 192, p. 167-184.

Doerner, J. P., and Carrara, P. E., 2001, Late Quaternary vegetation and climatic history of the Long Valley Area, west-central Idaho, U.S.A.: Quaternary Research, v. 56, p. 103-111.

Doner, L. A., 2009, A 19,000-year vegetation and climate record for Bear Lake, Utah and Idaho, in Rosenbaum, J. G., and Kaufman, D. S., eds., Paleoenvironments of Bear Lake, Utah and Idaho, and its catchment. Geological Society of America Special Paper 450, p. 213–223.

Gosse, J. C., Evenson, E. B., Klein, J., Lawn, B., and Middleton, R., 1995, Precise cosmogenic ^{10}Be measurements in western North America: Support for a global Younger Dryas cooling event: Geology, v. 23, no. 10, p. 877-880.

Haynes, C. V. Jr., McFaul, M., Brunswig, R. H., and Hopkins, K. D., 1998, Kersey-Kuner terrace investigations at the Dent and Bernhardt sites, Colorado: Geoarchaeology, v. 13, no. 2, p. 201-218.

Jackson, S. T., Betancourt, J. L., Lyford, M. E., Gray, S. T., and Rylander, K. A., 2005, A 40,000-year woodrat-midden record of vegetational and biogeographical dynamics in north-eastern Utah, USA: Journal of Biogeography, v. 32, p. 1085-1106.

Jiménez-Moreno, G., Fawcett, P. J., and Anderson, R. S., 2008, Millennial- and centennial-scale vegetation and climate changes during the late Pleistocene and Holocene from northern New Mexico (USA): Quaternary Science Reviews, v. 27, p. 1442-1452.

Johnson, B. G., Eppes, M. C., Diemer, J. A., Jiménez-Moreno, G., and Layzell, A. L., 2011, Post-glacial landscape response to climate variability in the southeastern San Juan Mountains of Colorado, USA: Quaternary Research, v. 76, p. 352-362.

Johnson, B. G., Jiménez-Moreno, G., Eppes, M. C., Diemer, J. A., and Stone, J. R., 2013, A multiproxy record of postglacial climate variability from a shallowing, 12-m deep sub-alpine bog in the southeastern San Juan Mountains of Colorado, USA: The Holocene, v. 23, no. 7, p. 1928-1938.

Krause, T. R., and Whitlock, C., 2013, Climate and vegetation change during the late-glacial/early-Holocene transition inferred from multiple proxy records from Blacktail Pond, Yellowstone National Park, USA: Quaternary Research, v. 79, p. 391-402.

Licciardi, J. M., and Pierce, K. L., 2008, Cosmogenic exposure-age chronologies of Pinedale and Bull Lake glaciations in greater Yellowstone and the Teton Range, USA: Quaternary Science Reviews, v. 27, p. 814-831.

Louderback, L. A., Rhode, D., Madsen, D. B., and Metcalf, M., 2015, Rapid vegetation shifts in the Uinta Mountains (Utah and Wyoming, USA) during the late Pleistocene and Holocene: Palaeogeography, Palaeoclimatology, Palaeoecology, v. 438, p. 327-343.

Lundeen, Z. J., and Brunelle, A., 2016, A 14,000-year record of fire, climate, and vegetation from the Bear River Range, southeast Idaho, USA: The Holocene, v. 26, no. 6, p. 833-842.

Marcott, S. A., Clark, P. U., Shakun, J. D., Brook, E. J., Davis, P. T., and Caffee, M. W., 2019, ^{10}Be age constraints on latest Pleistocene and Holocene cirque glaciation across the western United States: Climate and Atmospheric Science, v. 2, no. 5, doi: 10.1038/s41612-41019-40062-z.

Menounos, B., and Reasoner, M. A., 1997, Evidence for cirque glaciation in the Colorado Front Range during the Younger Dryas chronozone: Quaternary Research, v. 48, p. 38-47.

Minckley, T.A., Clementz, M., Kornfeld, M., Larson, M.L., and Finley, J.B., 2020, Late Pleistocene environments of the Bighorn Basin, Wyoming-Montana, USA: Quaternary Research, v. 99, p. 128-141.

Mumma, S. A., Whitlock, C., and Pierce, K., 2012, A 28,000 year history of vegetation and climate from Lower Red Rock Lake, Centennial Valley, southwestern Montana, USA: Palaeogeography, Palaeoclimatology, Palaeoecology, v. 326-328, p. 30-41.

Munroe, J. S., 2002, Timing of postglacial cirque reoccupation in the northern Uinta Mountains, northeastern Utah, U.S.A.: Arctic, Antarctic, and Alpine Research, v. 34, no. 1, p. 38-48.

Osborn, G., and Gerloff, L., 1997, Latest Pleistocene and early Holocene fluctuations of glaciers in the Canadian and Northern American Rockies: Quaternary International, v. 38/39, p. 7-19.

Pribyl, P., and Shuman, B. J., 2014, A computational approach to Quaternary lake-level reconstruction applied in the central Rocky Mountains, Wyoming, USA: Quaternary Research, v. 82, p. 249-259.

Reasoner, M. A., and Huber, U. M., 1999, Postglacial palaeoenvironments of the upper Bow Valley, Banff National Park, Alberta, Canada: Quaternary Science Reviews, v. 18, p. 475-492.

Reasoner, M. A., and Jodry, M. A., 2000, Rapid response of alpine timberline vegetation to the Younger Dryas climate oscillation in the Colorado Rocky Mountains, USA: Geology, v. 28, no. 1, p. 51-54.

Reasoner, M. A., Osborn, G., and Rutter, N. W., 1994, Age of the Crowfoot advance in the Canadian Rocky Mountains: A glacial event coeval with the Younger Dryas oscillation: Geology, v. 22, p. 439-442.

Rosenbaum, J. G., and Heil, C. W., Jr., 2009, The glacial/deglacial history of sedimentation in Bear Lake, Utah and Idaho, in Rosenbaum, J. G., and Kaufman, D. S., eds., Paleoenvironments of Bear Lake, Utah and Idaho, and its catchment. Geological Society of America Special Paper 450, p. 247-261.

Schachtman, N. S., MacGregor, K. R., Myrbo, A., Hencir, N. R., Riihimaki, C. A., Thole, J. T., and Bradtmiller, L. I., 2015, Lake core record of Grinnell Glacier dynamics during the latest Pleistocene deglaciation and the Younger Dryas, Glacier National Park, Montana, USA: Quaternary Research, v. 84, p. 1-11.

Shuman, B., Henderson, A. K., Colman, S. M., Stone, J. R., Fritz, S. C., Stevens, L. R., Power, M. J., and Whitlock, C., 2009, Holocene lake-level trends in the Rocky Mountains, U.S.A.: Quaternary Science Reviews, v. 28, p. 1861-1879.

Shuman, B., Pribyl, P., Minckley, T. A., and Shinker, J. J., 2010, Rapid hydrologic shifts and prolonged droughts in Rocky Mountain headwaters during the Holocene: Geophysical Research Letters, v. 37, p. L06701, doi: 06710.01029/02009GL042196,.

Shuman, B. N., Pribyl, P., and Buettner, J., 2015, Hydrologic changes in Colorado during the mid-Holocene and Younger Dryas: Quaternary Research, v. 84, p. 187-199.

Toney, J. L., and Anderson, R. S., 2006, A postglacial palaeoecological record from the San Juan Mountains of Colorado USA: fire, climate and vegetation history: The Holocene, v. 16, no. 4, p. 505-517.

Eastern Pacific Ocean

Addison, J. A., Finney, B. P., Dean, W. E., Davies, M. H., Mix, A. C., Stoner, J. S., and Jaeger, J. M., 2012, Productivity and sedimentary $\delta^{15}N$ variability for the last 17,000 years along the northern Gulf of Alaska continental slope: Paleoceanography, v. 27, p. PA1206, doi: 10.1029/2011PA002161.

Barron, J. A., Bukry, D., Dean, W. E., Addison, J. A., and Finney, B., 2009, Paleoceanography of the Gulf of Alaska during the past 15,000 years: Results from diatoms, silicoflagellates, and geochemistry: Marine Micropaleontology, v. 72, p. 176-195.

Barron, J. A., Heusser, L., Herbert, T., and Lyle, M., 2003, High-resolution climatic evolution of coastal northern California during the past 16,000 years: Paleoceanography, v. 18, no. 1, p. 1020, doi: 10.1029/2002PA000768.

Cannariato, K. G., Kennett, J. P., and Behl, R. J., 1999, Biotic response to late Quaternary rapid climate switches in Santa Barbara Basin: Ecological and evolutionary implications: Geology, v. 27, no. 1, p. 63-66.

Davies, M. H., Mix, A. C., Stoner, J. S., Addison, J. A., Jaeger, J., Finney, B., and Wiest, J., 2011, The deglacial transition on the southeastern Alaska Margin: Meltwater input, sea level rise, marine

productivity, and sedimentary anoxia: Paleoceanography, v. 26, p. PA2223, doi: 2210.1029/2010PA002051.

Emmer, E., and Thunnell, R. C., 2000, Nitrogen isotope variations in Santa Barbara Basin sediments: Implications for denitrification in the eastern tropical North Pacific during the last 50,000 years: Paleoceanography, v. 15, no. 4, p. 377-387.

Hendy, I. L., 2010, The paleoclimatic response of the Southern Californian Margin to the rapid climate change of the last 60 ka: A regional overview: Quaternary International, v. 215, p. 62-73.

Hendy, I. L., Kennett, J. P., Roark, E. B., and Ingram, B. L., 2002, Apparent synchroneity of submillennial scale climate events between Greenland and Santa Barbara Basin, California from 30-10 ka: Quaternary Science Reviews, v. 21, p. 1167-1184.

Heusser, L. E., and Sirocko, F., 1997, Millennial pulsing of environmental change in southern California from the past 24 k.y.: A record of Indo-Pacific ENSO events?: Geology, v. 25, no. 3, p. 243-246.

Hill, T. M., Kennett, J. P., Pak, D. K., Behl, R. J., Robert, C., and Beaufort, R. L., 2006, Pre-Bølling warming in Santa Barbara Basin, California: surface and intermediate water records of early deglacial warmth: Quaternary Science Reviews, v. 25, p. 2835-2845.

Hinrichs, K. -U., Rinna, J., Rullkötter, J., and Stein, R., 1997, A 160-kyr record of alkenone-derived sea-surface temperatures from Santa Barbara Basin sediments: Naturwissenschaften, v. 84, p. 126-128.

Keigwin, L. D., 2002, Late Pleistocene-Holocene paleoceanography and ventilation of the Gulf of California: Journal of Oceanography, v. 58, p. 421-432.

Kennett, J. P., and Ingram, B. L., 1995, A 20,000-year record of ocean circulation and climate change from the Santa Barbara Basin: Nature, v. 377, p. 510-514.

Kienast, S. S., and McKay, J. L., 2001, Sea surface temperatures in the subarctic Northeast Pacific reflect millennial-scale climate oscillations during the last 16 kyrs: Geophysical Research Letters, v. 28, no. 8, p. 1563-1566.

Mix, A. C., Lund, D. C., Pisias, N. G., Bodén, P., Bornmalm, L., Lyle, M., and Pike, J., 1999, Rapid climate oscillations in the Northeast Pacific during the last deglaciation reflect Northern and Southern Hemisphere sources, in Clark, P. U., Webb, S., and Keigwin, D., eds., Mechanisms of Global Climate Change at Millennial Time Scales. Geophysical Monograph Series, vol. 112, p. 127-148.

Nederbragt, A. J., and Thurow, J., 2005, Amplitude of ENSO cycles in the Santa Barbara Basin, off California, during the past 15 000 years: Journal of Quaternary Science, v. 20, no. 5, p. 447-456.

Pak, D. K., Lea, D. W., and Kennett, J. P., 2012, Millennial scale changes in sea surface temperature and ocean circulation in the northeast Pacific, 10–60 kyr BP: Paleoceanography, v. 27, p. PA1212, doi: 1210.1029/2011PA002238.

Patterson, R. T., 1993, Late Quaternary benthic foraminiferal biofacies and paleoceanography of Queen Charlotte Sound and Southern Hecate Strait, British Columbia: Journal of Foraminiferal Research, v. 23, no. 1, p. 1-18.

Pisias, N. G., Mix, A. C., and Heusser, L., 2001, Millennial scale climate variability of the northeast Pacific Ocean and northwest North America based on radiolaria and pollen: Quaternary Science Reviews, v. 20, p. 1561-1576.

Praetorius, S. K., and Mix, A. C., 2014, Synchronization of North Pacific and Greenland climates preceded abrupt deglacial warming: Science, v. 6195, p. 444-448.

Praetorius, S. K., Mix, A. C., Walczak, M. H., Wolhowe, M. D., Addison, J. A., and Prahl, F. G., 2015, North Pacific deglacial hypoxic events linked to abrupt ocean warming: Nature, v. 527, p. 362-366.

Roark, E. B., Ingram, B. L., Southon, J., and Kennett, J. P., 2003, Holocene foraminiferal radiocarbon record of paleocirculation in the Santa Barbara Basin: Geology, v. 31, no. 4, p. 379-382.

Seki, O., Ishiwatari, R., and Matsumoto, K., 2002, Millennial climate oscillations in NE Pacific surface waters over the last 82 kyr: New evidence from alkenones: Geophysical Research Letters, v. 29, no. 23, p. 2144, doi: 2110.1029/2002GL015200.

van Geen, A., Fairbanks, R. G., Dartnell, P., McGann, M., Gardner, J. V., and Kashgarian, M., 1995, Ventilation changes in the northeast Pacific during the last deglaciation: Paleoceanography, v. 11, no. 5, p. 519-528.

Gulf of California

Álvarez, M. C., Flores, J. A., Sierro, F. J., and Molina-Cruz, A., 2010, The coccolithophore record for the last 11 000 years in the Gulf of California: Journal of Marine Systems, v. 80, p. 184-190.

Arellano-Torres, E., Mora-Rivera, A. J., Vázquez-Romero, P., Nava-Sánchez, E. H., Kasper-Zubillaga, J. J., and Lozano-García, M. S., 2020, Comparisons between marine productivity and terrestrial input records in the Gulf of California over the last 28 ka: Journal of Quaternary Science, v. 35, no. 3, p. 479-491.

Barron, J. A., Bukry, D., and Dean, W. E., 2005, Paleoceanographic history of the Guaymas Basin, Gulf of California, during the past 15,000 years based on diatoms, silicoflagellates, and biogenic sediments: Marine Micropaleontology, v. 56, p. 81-102.

Barron, J. A., Bukry, D., and Bischoff, J. L., 2004, High resolution paleoceanography of the Guaymas Basin, Gulf of California, during the past 15 000 years: Marine Micropaleontology, v. 50, p. 185-207.

Cheshire, H., Thurow, J., and Nederbragt, A. J., 2005, Late Quaternary climate change record from two long sediment cores from Guaymas Basin, Gulf of California: Journal of Quaternary Science, v. 20, p. 457-469.

Dean, W. E., 2006, The geochemical record of the last 17,000 years in the Guaymas Basin, Gulf of California: Chemical Geology, v. 232, p. 87-98.

Keigwin, L. D., and Jones, G. A., 1990, Deglacial climatic oscillations in the Gulf of California: Paleoceanography, v. 5, no. 6, p. 1009-1023.

McClymont, E. L., Ganeshram, R. S., Pichevin, L. E., Talbot, H. M., van Dongen, B. E., Thunell, R. C., Haywood, A. M., Singarayer, J. S., and Valdes, P. J., 2012, Sea-surface temperature records of Termination 1 in the Gulf of California: Challenges for seasonal and interannual analogues of tropical Pacific climate change: Paleoceanography, v. 27, p. PA2202, doi: 2210.1029/2011PA002226.

Pichevin, L., Ganeshram, R. S., Reynolds, B. C., Prahl, F., Pedersen, T. F., Thunell, R., and McClymont, E. L., 2012, Silicic acid biogeochemistry in the Gulf of California: Insights from sedimentary Si isotopes: Paleoceanography, v. 27, p. PA2201, doi: 2210.1029/2011PA002237.

Price, A. M., Mertens, K. N., Pospelova, V., Pedersen, T. F., and Ganeshram, R. S., 2013, Late Quaternary climatic and oceanographic changes in the Northeast Pacific as recorded by dinoflagellate cysts from Guaymas Basin, Gulf of California (Mexico): Paleoceanography, v. 28, p. 200-212.

Pride, C., Thunnell, R., Sigman, D., Keigwin, L., and Altabet, M., 1999, Nitrogen isotopic variations in the Gulf of California since the last deglaciation: Response to global climate change: Paleoceanography, v. 14, no. 3, p. 397-409.

Sancetta, C., 1995, Diatoms in the Gulf of California: Seasonal flux patterns and the sediment record for the last 15,000 years: Paleoceanography, v. 10, no. 1, p. 67-84.

Mexico

Heine, K., 1994, The late-glacial moraine sequences in Mexico: is there evidence for the Younger Dryas event?: Palaeogeography, Palaeoclimatology, Palaeoecology, v. 112, p. 113-123.

Metcalfe, S. A., Bimpson, A., Courtice, A. J., O'Hara, S. L., and Taylor, D. M., 1997, Climate change at the monsoon/Westerly boundary in Northern Mexico: Journal of Paleolimnology, v. 17, p. 155-171.

Metcalfe, S. A., O'Hara, S. L., Caballero, M., and Davies, S. J., 2000, Records of Late Pleistocene-Holocene climatic change in Mexico - a review: Quaternary Science Reviews, v. 19, p. 699-721.

Ortega-Ramírez, J. R., Valiente-Banuet, A., Urrutia-Fucugauchi, J., Mortera-Gutiérrez, C. A., and Alvarado-Valdez, G., 1998, Paleoclimatic changes during the Late Pleistocene – Holocene in Laguna

Babícora, near the Chihuahuan Desert, México: Canadian Journal of Earth Sciences, v. 35, p. 1168-1179.
Rhode, D., 2002, Early Holocene juniper woodland and chaparral taxa in the central Baja California Peninsula, Mexico: Quaternary Research, v. 57, p. 102-108.
Roy, P. D., Chávez-Lara, C. M., Beramendi-Orosco, L. E., Sánchez-Zavala, J. L., Muthu-Sankar, G., Lozano-Santacruz, R., Quiroz-Jimenez, J. D., and López-Balbiaux, N., 2015, Paleohydrology of the Santiaguillo Basin (Mexico) since late last glacial and climate variation in southern part of western subtropical North America: Quaternary Research, v. 84, p. 335-347.
Roy, P. D., Jonathan, M. P., Pérez-Cruz, L. L., Sánchez-Córdova, M.M., Quiroz-Jiménez, J. D., and Romero, F. M., 2012, A millennial-scale Late Pleistocene–Holocene palaeoclimatic record from the western Chihuahua Desert, Mexico: Boreas, v. 41, p. 707-717.
Roy, P. D., Quiroz-Jiménez, J. D., Pérez-Cruz, L. L., Lozano-García, S., Metcalfe, S. E., Lozano-Santacruz, R., López-Balbiaux, N., Sánchez-Zavala, J. L., and Romero, F. M., 2013, Late Quaternary paleohydrological conditions in the drylands of northern Mexico: a summer precipitation proxy record of the last 80 cal ka BP: Quaternary Science Reviews, v. 78, p. 342-354.

Pacific Northwest

Beck, C. W., Bryant, V. M., and Jenkins, D. L., 2018, Analysis of Younger Dryas–Early Holocene pollen in sediments of Paisley Cave 2, south-central Oregon: Palynology, v. 42, no. 2, p. 168-179.
Beck, C. W., Bryant, V. M., and Jenkins, D. L., 2020, Comparison of *Neotoma* (packrat) feces to associated sediments from Paisley Caves, Oregon, U.S.A.: Palynology, v. 44, no. 4, p. 723-741.
Briles, C. E., Whitlock, C., and Bartlein, P. J., 2005, Postglacial vegetation, fire, and climate history of the Siskiyou Mountains, Oregon, USA: Quaternary Research, v. 64, p. 44-56.
Friele, P. A., and Clague, J. J., 2002, Younger Dryas readvance in Squamish river valley, southern Coast mountains, British Columbia: Quaternary Science Reviews, v. 21, p. 1925-1933.
Galloway, J. M., Patterson, R. T., Doherty, C. T., and Roe, H. M., 2007, Multi-proxy evidence of postglacial climate and environmental change at Two Frog Lake, central mainland coast of British Columbia, Canada: Journal of Paleolimnology, v. 38, p. 569-588.
Gilmour, D. M., Butler, V. L., O'Connor, J. E., Davis, E. B., Culleton, B. J., Kennett, D. J., and Hodgins, G., 2015, Chronology and ecology of late Pleistocene megafauna in the northern Willamette Valley, Oregon: Quaternary Research, v. 83, p. 127-136.
Grigg, L. D., and Whitlock, C., 1998, Late-glacial vegetation and climate change in western Oregon: Quaternary Research, v. 49, p. 287-298.
Heine, J. T., 1998, Extent, timing, and climatic implications of glacier advances Mount Rainier, Washington, U.S.A., at the Pleistocene/Holocene transition: Quaternary Science Reviews, v. 17, p. 1139-1148.
Hudson, A. M., Hatchett, B. J., Quade, J., Boyle, D. P., Bassett, S. D., Ali, G., and DelosSantos, M. G., 2019, North-south dipole in winter hydroclimate in the western United States during the last deglaciation, v. 9, p. 4826: 4810.1038/s41598-41019-41197-y.
Kovanen, D. J., and Easterbrook, D. J., 2002, Timing and extent of Allerød and Younger Dryas age (ca. 12,500–10,000 ^{14}C yr B.P.) oscillations of the Cordilleran Ice Sheet in the Fraser Lowland, western North America: Quaternary Research, v. 57, p. 208-224.
Kovanen, D. J., and Slaymaker, O., 2005, Fluctuations of the Deming Glacier and theoretical equilibrium line altitudes during the Late Pleistocene and Early Holocene on Mount Baker, Washington, USA: Boreas, v. 34, p. 157-175.
Lacourse, T., 2005, Late Quaternary dynamics of forest vegetation on northern Vancouver Island, British Columbia, Canada: Quaternary Science Reviews, v. 24, p. 105-121.

Lacourse, T., Delepine, J. M., Hoffman, E. H., and Mathewes, R. W., 2012, A 14,000 year vegetation history of a hypermaritime island on the outer Pacific coast of Canada based on fossil pollen, spores and conifer stomata: Quaternary Research, v. 78, p. 572-582.

Lacourse, T., Mathewes, R. W., and Fedje, D. W., 2005, Late-glacial vegetation dynamics of the Queen Charlotte Islands and adjacent continental shelf, British Columbia, Canada: Palaeogeography, Palaeoclimatology, Palaeoecology, v. 226, p. 36-57.

Licciardi, J. M., Clark, P. U., Brook, E. J., Elmore, D., and Sharma, P., 2004, Variable responses of western U.S. glaciers during the last deglaciation: Geology, v. 32, no. 1, p. 81-84.

Mann, D. H., and Hamilton, T. D., 1995, Late Pleistocene and Holocene paleoenvironments of the North Pacific coast: Quaternary Science Reviews, v. 14, p. 449-471.

Mathewes, R. W., 1993, Evidence for a Younger Dryas-age cooling event on the British Columbia coast: Quaternary Science Reviews, v. 12, p. 321-331.

Mathewes, R. W., Heusser, L. E., and Patterson, R. T., 1993, Evidence for a Younger Dryas-like cooling event on the British Columbia coast: Geology, v. 21, p. 101-104.

Patterson, R. T., Guilbault, J.-P., Thomson, R. E., and Luternauer, J. L., 1995, Foraminiferal evidence of Younger Dryas age cooling on the British Columbia shelf: Géographie physique et Quaternaire, v. 49, no. 3, p. 409-427.

Pellatt, M. G., Mathewes, R. W., and Clague, J. J., 2002, Implications of a late-glacial pollen record for the glacial and climatic history of the Fraser Lowland, British Columbia: Palaeogeography, Palaeoclimatology, Palaeoecology, v. 180, p. 147-157.

Sea, D. S., and Whitlock, C., 1995, Postglacial vegetation and climate of the Cascade Range, central Oregon: Quaternary Research, v. 43, p. 370-381.

Vacco, D. A., Clark, P. U., Mix, A. C., Cheng, H., and Edwards, R. L., 2005, A speleothem record of Younger Dryas cooling, Klamath Mountains, Oregon, USA: Quaternary Research, v. 64, p. 249-256.

Wilcox, P. S., Dorale, J. A., Baichtal, J. F., Spötl, C., Fowell, S. J., Edwards, R. L., and Kovarik, J. L., 2019, Millennial-scale glacial climate variability in southeastern Alaska follows Dansgaard-Oeschger cyclicity: Nature Scientific Reports, v. 9, p. 7880; doi: 7810.1038/s41598-41019-44231-41591.

Worona, M. A., and Whitlock, C., 1995, Late Quaternary vegetation and climate history near Little Lake, central Coast Range, Oregon: Geological Society of America Bulletin, v. 107, no. 7, p. 867-876.

Southwestern USA

Adams, K. D., Goebel, T., Graf, K., Smith, G. M., Camp, A. J., Briggs, R. W., and Rhode, D., 2008, Late Pleistocene and early Holocene lake-level fluctuations in the Lahontan Basin, Nevada: Implications for the distribution of archaeological sites: Geoarchaeology, v. 23, no. 5, p. 608-643.

Adams, K. D., and Rhodes, E. J., 2019, Late Pleistocene to present lake-level fluctuations at Pyramid and Winnemucca lakes, Nevada, USA: Quaternary Research, v. 92, p. 146-164.

Anderson, R. Y., Allen, B. D., and Menking, K. M., 2002, Geomorphic expression of abrupt climate change in southwestern North America at the Glacial Termination: Quaternary Research, v. 57, p. 371-381.

Asmerom, Y., Polyak, V., Burns, S., and Rassmussen, J., 2007, Solar forcing of Holocene climate: New insights from a speleothem record, southwestern United States: Geology, v. 35, no. 1, p. 1-4.

Asmerom, Y., Polyak, V. J., and Burns, S. J., 2010, Variable winter moisture in the southwestern United States linked to rapid glacial climate shifts: Nature Geoscience, v. 3, p. 114-117.

Asmerom, Y., Polyak, V. J., and Lachniet, M., 2017, Extrapolar climate reversal during the last deglaciation: Nature Scientific Reports, v. 17, no. 7, p. 10.1038/s41598-41017-07721-41598.

Bacon, S. N., Burke, R. M., Pezzopane, S. K., and Jayko, A. S., 2006, Last glacial maximum and Holocene lake levels of Owens Lake, eastern California, USA: Quaternary Science Reviews, v. 25, p. 1264-1282.

Balakrishnan, M., Yapp, C. J., Meltzer, D. J., and Theler, J. L., 2005, Paleoenvironment of the Folsom archaeological site, New Mexico, USA, approximately 10,500 ^{14}C yr B.P. as inferred from the stable isotope composition of fossil land snail shells: Quaternary Research, v. 63, p. 31-44.

Ball, G. I., Noble, P. J., Stephens, B. M., Higgins, A., Mensing, S. A., and Aluwihare, L. I., 2018, A lignin, diatom, and pollen record spanning the Pleistocene-Holocene transition at Fallen Leaf Lake, Sierra Nevada, California, USA, in Starratt, S. W., and Rosen, M. R., eds., From Saline to Freshwater: The Diversity of Western Lakes in Space and Time: Geological Society of America Special Paper 536, p. 1–18, doi: 10.1130/2018.2536(01).

Ballenger, J. A. M., Holliday, V. T., Kowler, A. L., Reitze, W. T., Prasciunas, M. M., Miller, D. S., and Windingstad, J. D., 2011, Evidence for Younger Dryas global climate oscillation and human response in the American Southwest: Quaternary International, v. 242, p. 502-519.

Bell, J. W., Brune, J. N., Liu, T., Zreda, M., and Yount, J. C., 1998, Dating precariously balanced rocks in seismically active parts of California and Nevada: Geology, v. 26, no. 6, p. 495-498.

Benson, L., Burdett, J., Lund, S., Kashgarian, M., and Mensing, S., 1997, Nearly synchronous climate change in the Northern Hemisphere during the last glacial termination: Nature, v. 388, p. 263-265.

Benson, L., Currey, D., Lao, Y., and Hostetler, S., 1992, Lake-size variations in the Lahontan and Bonneville basins between 13,000 and 9000 ^{14}C yr B.P.: Palaeogeography, Palaeoclimatology, Palaeoecology, v. 95, p. 19-32.

Benson, L., Kashgarian, M., and Rubin, M., 1995, Carbonate deposition, Pyramid Lake subbasin, Nevada: 2. Lake levels and polar-jet stream positions reconstructed from radiocarbon ages and elevations of carbonates (tufas) deposited in the Lahontan Basin: Palaeogeography Palaeoclimatology Palaeoecology, v. 117, p. 1-30.

Benson, L. V., Currey, D. R., Dorn, R. I., Lajoie, K. R., Oviatt, C. G., Robinson, S. W., Smith, G. I., and Stine, S., 1990, Chronology of expansion and contraction of four Great Basin lake systems during the past 35,000 years: Palaeogeography, Palaeoclimatology, Palaeoecology, v. 78, p. 241-286.

Benson, L. V., Lund, S. P., Smoot, J. P., Rhode, D. E., Spencer, R. J., Verosub, K. L., Louderback, L. A., Johnson, C. A., Rye, R. O., and Negrini, R. M., 2011, The rise and fall of Lake Bonneville between 45 and 10.5 ka: Quaternary International, v. 235, p. 57-69.

Benson, L. V., Smoot, J. P., Lund, S. P., Mensing, S. A., Foit, F.F., Jr., and Rye, R. O., 2013, Insights from a synthesis of old and new climate-proxy data from the Pyramid and Winnemucca lake basins for the period 48 to 11.5 cal ka: Quaternary International, v. 310, p. 62-82.

Briggs, R. W., Wesnousky, S. G., and Adams, K. D., 2005, Late Pleistocene and late Holocene lake highstands in the Pyramid Lake subbasin of Lake Lahontan, Nevada, USA: Quaternary Research, v. 64, p. 257-263.

Brook, G. A., Ellwood, B. B., Railsback, L. B., and Cowart, J. B., 2006, A 164 ka record of environmental change in the American Southwest from a Carlsbad Cavern speleothem: Palaeogeography, Palaeoclimatology, Palaeoecology, v. 237, p. 483-507.

Cisneros-Dozal, L. M., Heikoop, J. M., Fessenden, J., Anderson, R. S., Meyers, P. A., Allen, C. D., Hess, M., Larson, T., Perkins, G., and Rearick, M., 2010, A 15 000-year record of climate change in northern New Mexico, USA, inferred from isotopic and elemental contents of bog sediments: Journal of Quaternary Science, v. 25, p. 1001-1007.

Cole, K. L., and Arundel, S. T., 2005, Carbon isotopes from fossil packrat pellets and elevational movements of Utah agave plants reveal the Younger Dryas cold period in Grand Canyon, Arizona: Geology, v. 33, no. 9, p. 713-716.

Goebel, T., Hockett, B., Adams, K. D., Rhode, D., and Graf, K., 2011, Climate, environment, and humans in North America's Great Basin during the Younger Dryas, 12,900-11,600 calendar years ago: Quaternary International, v. 242, p. 479-501.

Hall, S. A., and Penner, W. L., 2013, Stable carbon isotopes, C_3–C_4 vegetation, and 12,800 years of climate change in central New Mexico, USA: Palaeogeography, Palaeoclimatology, Palaeoecology, v. 369, p. 272-281.

Hall, S. A., Penner, W. L., Palacios-Fest, M. R., Metcalfe, A. L., and Smith, S. J., 2012, Cool, wet conditions late in the Younger Dryas in semi-arid New Mexico: Quaternary Research, v. 77, p. 87-95.

Harris-Parks, E., 2016, The micromorphology of Younger Dryas-aged black mats from Nevada, Arizona, Texas and New Mexico: Quaternary Research, v. 85, p. 94-106.

Haynes, C. V., Jr., 1991, Geoarchaeological and paleohydrological evidence for a Clovis-age drought in North America and its bearing on extinction: Quaternary Research, v. 35, p. 438-450.

Haynes, C. V., Jr., 2008, Younger Dryas "black mats" and the Rancholabrean termination in North America: Proceedings of the National Academy of Sciences USA, v. 105, no. 18, p. 6520-6525.

Heusser, L. E., Kirby, M. E., and Nichols, J. E., 2015, Pollen-based evidence of extreme drought during the last glacial (32.6-9.0 ka) in coastal southern California: Quaternary Science Reviews, v. 126, p. 242-253.

Holliday, V. T., 2000, Folsom drought and episodic drying on the Southern High Plains from 10,900–10,200 ^{14}C yr B.P.: Quaternary Research, v. 53, p. 1-12.

Holliday, V. T., Huckell, B. B., Mayer, J. H., Forman, S. L., and McFadden, L. D., 2006, Geoarchaeology of the Boca Negra Wash area, Albuquerque Basin, New Mexico, USA: Geoarchaeology, v. 21, no. 8, p. 765-802.

Holmgren, C. A., Betancourt, J. L., and Rylander, K. A., 2006, A 36,000-yr vegetation history from the Peloncillo Mountains, southeastern Arizona, USA: Palaeogeography, Palaeoclimatology, Palaeoecology, v. 240, p. 405-422.

Honke, J. S., Pigati, J. S., Wilson, J., Bright, J., Goldstein, H. L., Skipp, G. L., Reheis, M. C., and Havens, J. C., 2019, Late Quaternary paleohydrology of desert wetlands and pluvial lakes in the Soda Lake basin, central Mojave Desert, California (USA): Quaternary Science Reviews, v. 216, p. 89-106.

Huckleberry, G., Beck, C., Jones, G. T., Holmes, A., Cannon, M., Livingston, S., and Broughton, J. M., 2001, Terminal Pleistocene/early Holocene environmental change at the Sunshine Locality, north-central Nevada, U.S.A.: Quaternary Research, v. 55, p. 303-312.

Ibarra, D. E., Egger, A. E., Weaver, K. L., Harris, C. R., and Maher, K., 2014, Rise and fall of late Pleistocene pluvial lakes in response to reduced evaporation and precipitation: Evidence from Lake Surprise, California: Geological Society of America Bulletin, v. 126, no. 11/12, p. 1387-1415.

Kirby, M. E., Feakins, S. J., Bonuso, N., Fantozzi, J. M., and Hiner, C. A., 2013, Latest Pleistocene to Holocene hydroclimates from Lake Elsinore, California: Quaternary Science Reviews, v. 76, p. 1-15.

Kirby, M. E., Heusser, L., Scholz, C., Ramezan, R., Anderson, M. A., Markel, B., Rhodes, E., Glover, K. C., Fantozzi, J., Hiner, C., Price, B., and Rangel, H., 2018, A late Wisconsin (32–10k cal a BP) history of pluvials, droughts and vegetation in the Pacific south-west United States (Lake Elsinore, CA): Journal of Quaternary Science, v. 33, no. 2, p. 238-254.

Kirby, M. E., Knell, E. J., Anderson, W. T., Lachniet, M. S., Palermo, J., Eeg, H., Lucero, R., Murrieta, R., Arevalo, A., Silveira, E., and Hiner, C. A., 2015, Evidence for insolation and Pacific forcing of late glacial through Holocene climate in the central Mojave Desert (Silver Lake, CA): Quaternary Research, v. 84, p. 174-186.

Lachniet, M. S., Denniston, R. F., Asmerom, Y., and Polyak, V. J., 2014, Orbital control of western North America atmospheric circulation and climate over two glacial cycles: Nature Communications, v. 5, p. 3805, doi: 3810.1038/ncomms4805.

Leidelmeijer, J.A., Kirby, M.E., MacDonald, G., Carlin, J.A., Avila, J. Han, J., Nauman, B., Loyd, S., Nichols, K., Ramezan, R., 2021, Younger Dryas to early Holocene (12.9 to 8.1 ka) limnological and hydrological change at Barley Lake, California (northern California Coast Range). Quaternary Research, doi.org/10.1017/qua.2021.9.

Liu, T., Broecker, W. S., Bell, J. W., and Mandeville, C. W., 2000, Terminal Pleistocene wet event recorded in rock varnish from Las Vegas Valley, southern Nevada: Palaeogeography, Palaeoclimatology, Palaeoecology, v. 161, p. 423-433.

Love, D. W., Allen, B. D., Morgan, G. S., and Myers, R. G., 2014, Radiocarbon and fossil vertebrate ages of late Pleistocene and Holocene sediments imply rapid rates of evaporite deposition in the northern Tularosa Basin, south central New Mexico: New Mexico Geological Society Guidebook, 65th Field Conference, Geology of the Sacramento Mountains Region, p. 135-142.

Madsen, D. B., Rhode, D., Grayson, D. K., Broughton, J. M., Livingston, S. D., Hunt, J., Quade, J., Schmitt, D. N., and Shaver, M. W., III, 2001, Late Quaternary environmental change in the Bonneville basin, western USA: Palaeogeography, Palaeoclimatology, Palaeoecology, v. 167, p. 243-271.

MacDonald, G. M., Moser, K. A., Bloom, A. M., Porinchu, D. F., Potito, A. P., Wolfe, B. B., Edwards, T. W. D., Petel, A., Orme, A. R., and Orme, A. J., 2008, Evidence of temperature depression and hydrological variations in the eastern Sierra Nevada during the Younger Dryas stade: Quaternary Research, v. 70, p. 131-140.

Mann, D. H., and Meltzer, D. J., 2007, Millennial-scale dynamics of valley fills over the past 12,000 ^{14}C yr in northeastern New Mexico, USA: Geological Society of America Bulletin, v. 119, no. 11/12, p. 1433-1448.

McGee, D., Quade, J., Edwards, R. L., Broecker, W. S., Cheng, H., Reiners, P. W., and Evenson, N., 2012, Lacustrine cave carbonates: Novel archives of paleohydrologic change in the Bonneville Basin (Utah, USA): Earth and Planetary Science Letters, v. 351-352, p. 182-194.

Meltzer, D. J., and Holliday, V. T., 2010, Would North American Paleoindians have noticed Younger Dryas age climate changes?: Journal of World Prehistory, v. 23, p. 1-41.

Menking, K. M., Polyak, V. J., Anderson, R. Y., and Asmerom, Y., 2018, Climate history of the southwestern United States based on Estancia Basin hydrologic variability from 69 to 10 ka: Quaternary Science Reviews, v. 200, p. 237-252.

Mensing, S. A., 2001, Late-glacial and early Holocene vegetation and climate change near Owens Lake, eastern California: Quaternary Research, v. 55, p. 57-65.

Munroe, J. S., Bigl, M. F., Silverman, A. E., and Laabs, B. J. C., 2019, Records of late Quaternary environmental change from high-elevation lakes in the Ruby Mountains and East Humboldt Range, Nevada, in Starratt, S. W., and Rosen, M. R., eds., From Saline to Freshwater: The Diversity of Western Lakes in Space and Time, Volume Geological Society of America Special Paper 536, p. 1-20.

Munroe, J. S., and Laabs, B. J. C., 2013, Latest Pleistocene history of pluvial Lake Franklin, northeastern Nevada, USA: Geological Society of America Bulletin, v. 125, no. 3/4, p. 322-342.

Oster, J. L., Montañez, I. P., Santare, L. R., Sharp, W. D., Wong, C., and Cooper, K. M., 2015, Stalagmite records of hydroclimate in central California during termination 1: Quaternary Science Reviews, v. 127, p. 199-214.

Oster, J. L., Montañez, I. P., Sharp, W. D., and Cooper, K. M., 2009, Late Pleistocene California droughts during deglaciation and Arctic warming: Earth and Planetary Science Letters, v. 288, p. 434-443.

Oviatt, C. G., 1997, Lake Bonneville fluctuations and global climate change: Geology, v. 25, no. 2, p. 155-158.

Oviatt, C. G., Madsen, D. B., and Schmitt, D. N., 2003, Late Pleistocene and early Holocene rivers and wetlands in the Bonneville basin of western North America: Quaternary Research, v. 60, p. 200-210.

Oviatt, C. G., Miller, D. M., McGeehin, J. P., Zachary, C., and Mahan, S. A., 2005, The Younger Dryas phase of Great Salt Lake, Utah, USA: Palaeogeography, Palaeoclimatology, Palaeoecology, v. 219, p. 263-284.

Owen, L. A., Finkel, R. C., Minnich, R. A., and Perez, A. E., 2003, Extreme southwestern margin of late Quaternary glaciation in North America: Timing and controls: Geology, v. 31, no. 8, p. 729-732.

Palacios-Fest, M. R., and Holliday, V. T., 2018, Paleoecology of a ciénega at the Mockingbird Gap Site, Chupadera Draw, New Mexico: Quaternary Research, v. 89, p. 318-332.

Pigati, J. S., Miller, D. M., Bright, J. E., Mahan, S. A., Nekola, J. C., and Paces, J. B., 2011, Chronology, sedimentology, and microfauna of groundwater discharge deposits in the central Mojave Desert, Valley Wells, California: Geological Society of America Bulletin, v. 123, no. 11/12, p. 2224-2239.

Pigati, J. S., Springer, K. B., and Honke, J. S., 2019, Desert wetlands record hydrologic variability within the Younger Dryas chronozone, Mojave Desert, USA: Quaternary Research, v. 91, p. 51-62.

Polyak, V. J., Asmerom, Y., Burns, S. J., and Lachniet, M. S., 2012, Climatic backdrop to the terminal Pleistocene extinction of North American mammals: Geology, v. 40, no. 11, p. 1023-1026.

Polyak, V. J., Rasmussen, J. B. T., and Asmerom, Y., 2004, Prolonged wet period in the southwestern United States through the Younger Dryas: Geology, v. 32, no. 1, p. 5-8.

Porinchu, D. F., MacDonald, G. M., Bloom, A. M., and Moser, K. A., 2003, Late Pleistocene and early Holocene climate and limnological changes in the Sierra Nevada, California, USA inferred from midges (Insecta: Diptera: Chironomidae): Palaeogeography, Palaeoclimatology, Palaeoecology, v. 198, p. 403-422.

Quade, J., Forester, R. M., Pratt, W. L., and Carter, C., 1998, Black mats, spring-fed streams, and late-glacial-age recharge in the southern Great Basin: Quaternary Research, v. 49, p. 129-148.

Shurtliff, R. A., Nelson, S. T., McBride, J. H., Rey, K. A., Tucker, J. C., Godwin, S. B., and Tingey, D. G., 2017, A 13 000 year multi-proxy climate record from central Utah (western USA), emphasizing conditions leading to large mass movements: Boreas, v. 46, p. 308–324.

Springer, K. B., Manker, C. R., and Pigati, J. S., 2015, Dynamic response of desert wetlands to abrupt climate change: Proceedings of the National Academy of Sciences USA, v. 112, no. 47, p. 14522-14526.

Springer, K. B., Pigati, J. S., Manker, C. R., and Mahan, S. A., 2018, The Las Vegas Formation: U.S. Geological Survey Professional Paper 1839, 62 p., doi: 10.3133/pp1839.

Steponaitis, E., Andrews, A., McGee, D., Quade, J., Hsieh, Y.-T., Broecker, W. S., Shuman, B. N., Burns, S. J., and Cheng, H., 2015, Mid-Holocene drying of the U.S. Great Basin recorded in Nevada speleothems: Quaternary Science Reviews, v. 127, p. 174-185.

Street, J. H., Anderson, R. S., and Paytan, A., 2012, An organic geochemical record of Sierra Nevada climate since the LGM from Swamp Lake, Yosemite: Quaternary Science Reviews, v. 40, p. 89-106.

Wagner, J. D. M., Cole, J. E., Beck, J. W., Patchett, P. J., Henderson, G. M., and Barnett, H. R., 2010, Moisture variability in the southwestern United States linked to abrupt glacial climate change: Nature Geoscience, v. 3, p. 110-113.

Weng, C., and Jackson, S. T., 1999, Late glacial and Holocene vegetation history and paleoclimate of the Kaibab Plateau, Arizona: Palaeogeography, Palaeoclimatology, Palaeoecology, v. 153, p. 179-201.

Winograd, I. J., Landwehr, J. M., Coplen, T. B., Sharp, W. D., Riggs, A. C., Ludwig, K. R., and Kolesar, P. T., 2006, Devils Hole, Nevada, $\delta^{18}O$ record extended to the mid-Holocene: Quaternary Research, v. 66, p. 202-212.

Wurster, C. M., Patterson, W. P., McFarlane, D. A., Wassenaar, L. I., Hobson, K. A., Athfield, N. B., and Bird, M. I., 2008, Stable carbon and hydrogen isotopes from bat guano in the Grand Canyon, USA, reveal Younger Dryas and 8.2 ka events: Geology, v. 36, no. 9, p. 683-686.

SI 2a

Bonsai Lake, Alaska

Summary: Wilcox et al. (2020) used radiocarbon dating and measured pollen concentrations, organic content, C/N, $\delta^{13}C$, grain size, and magnetic susceptibility in sediments from a core taken at Bonsai Lake in southeastern Alaska to elucidate the nature and expression of the Younger Dryas (YD) climate event in the Pacific Northwest. They found the YD event consisted of at least two, and possibly three, distinct stages. The first stage was characterized by cooler and drier than modern conditions that prevailed between ~12.9 and 12.6 ka based on decreases in the percentages of pine and mountain hemlock pollen that were accompanied by increases in alder pollen and fern spores. The beginning of the second stage was marked by an increase in the percentages of pine and Sitka spruce which they interpreted as reflecting slightly warmer temperatures during the latter portion of the YD, between ~12.6 and 12.2 ka. Continued increasing temperatures and/or precipitation is indicated by a further increase in Sitka spruce and a concomitant decrease in pine between 12.2 and 11.7 ka.

Assessment: The pollen data from Bonsai Lake exhibit possible evidence of two stages within the YD climate event in southeastern Alaska with a transition at ~12.6, although we note that none of the other proxies show a marked change at this depth interval (their Figure 5). Although the data from Bonsai Lake data suggest a possible two-staged (or even three-staged) YD event, the lack of consistency between the pollen and all of the other proxies makes it difficult to determine the significance and magnitude of the inferred hydroclimate changes.

Citation: Wilcox, P. S., Fowell, S. J., and Baichtal, J. F., 2020, A mild Younger Dryas recorded in southeastern Alaska: Arctic, Antarctic, and Alpine Research, v. 50, p. 236-247.

Discovery Pond, Alaska

Summary: Kaufman et al. (2010) examined sediments from multiple cores taken from a small pond in southern Alaska that span the YD climate event. They used radiocarbon dating and measured concentrations of several climate proxies, including magnetic susceptibility, organic matter content, biogenic silica, pollen, and some microfossils, and found that temperatures in the region increased throughout the YD, reaching a maximum sometime around 11 ka. The data also exhibit a pronounced increase in the abundance of green algae at ~12.2 ka, which they attributed to a shift from wetland to open-water conditions driven by an increase in temperature and effective moisture during the second half of the YD.

Assessment: The proxy data from the Discovery Pond sediment cores clearly show an increase in green algae within their unit 2b, which they have bracketed in time between ~12.8 and 11.0 ka (their Figure 4). The authors interpret this increase to reflect warmer/wetter conditions beginning at ~12.2 ka, the timing of which was determined based on extrapolation of ages above and below unit 2b and assuming a constant sedimentation rate despite the climatic transition into and out of the YD climate event. Notably, none of the other proxies show a marked change at this depth interval. Although the data from Discovery Pond suggest the possibility of a two-staged YD event in this part of southern Alaska, the timing of the transition between stages is tenuous and the lack of consistency between the different proxies makes it difficult to determine the significance and magnitude of the inferred hydroclimate changes.

Citation: Kaufman, D. S., Anderson, R. S., Hub, F. S., Berg, E., and Werner, A., 2010, Evidence for a variable and wet Younger Dryas in southern Alaska: Quaternary Science Reviews, v. 29, p. 1445-1452.

Mount Waskey, Alaska

Summary: Young et al. (2019) used cosmogenic ^{10}Be to determine surface exposure ages of glacial moraine boulders (n=15), inboard erratic boulders (n=3), and an upvalley erratic boulder to determine the age of a late Pleistocene glacial advance near Mount Waskey in the Ahklun Mountains of southern Alaska. The ages indicate the glacier reached its maximum extent at 12.52±0.24 ka, early within the YD stadial, before retreating ~1 km during the remainder of the YD. The ages also showed the glacier remained at this upvalley position until 11.66±0.23 ka.

Assessment: The cosmogenic ^{10}Be ages show the glacier near Mount Waskey advanced during the early part of the YD, which was followed by glacial retreat through the remainder of the climate event. Although cosmogenic ages can be systematically offset from the true age because of issues related to production rates, exhumation, and episodic snow cover, the authors were careful in selecting boulders when sampling so they could avoid these issues as much as possible. Overall, the data presented by Young et al. indicate the two-stage character of the YD climate event extended into southern Alaska, although the exact timing of the transition between the cool/wet conditions required for glacial advancement and the warmer/drier conditions that led to glacial retreat can only be constrained to between ~12.5 and 11.7 ka.

Citation: Young, N. E., Briner, J. P., Schaefer, J., Zimmerman, S., and Finkel, R. C., 2019, Early Younger Dryas glacier culmination in southern Alaska: Implications for North Atlantic climate change during the last deglaciation: Geology, v. 47, p. 550-554.

Dove Springs Wash, Little Dixie Wash, and Mesquite Springs, California

Summary: Pigati et al. (2019) evaluated paleospring deposits exposed in outcrop at three sites in the Mojave Desert (Dove Springs Wash, Little Dixie Wash, and Mesquite Springs) as part of an effort to determine how fast spring ecosystems responded to past episodes of abrupt climate change. They used radiocarbon dating, grain size, redox properties of the sediments, organic content, and microfauna (terrestrial gastropods) to show that these ecosystems responded to climate change faster than they could delineate with ^{14}C dating.

Assessment: Hydrographs showing relative groundwater levels at each of the three sites (their Figure 5) clearly show conditions fluctuated between periods of high water table levels and active groundwater discharge represented by organic-rich black mats and times of low water table levels represented by aeolian, alluvial, or colluvial sediments that were deposited under dry conditions. Although the data demonstrate that unstable climate conditions prevailed during the YD event, the records do not contain evidence of two distinct stages within the event itself.

Citation: Pigati, J. S., Springer, K. B., and Honke, J. S., 2019, Desert wetlands record hydrologic variability within the Younger Dryas chronozone, Mojave Desert, USA: Quaternary Research, v. 91, p. 51-62.

Fallen Leaf Lake, California

Summary: Ball et al. (2018) examined core sediments dated by radiocarbon from Fallen Leaf Lake, California to reconstruct the response of the region’s aquatic and terrestrial ecosystems to climatic changes that accompanied the Younger Dryas, the end of the Pleistocene, and early Holocene warming. Lignin phenols indicate expansion of angiosperms at the Pleistocene-Holocene transition, whereas pollen shows closed canopy forests became more open and grasses and aster colonized meadows at this time. Within the YD event, a pulse of woody gymnosperm lignin was recorded at 13.0-12.6 ka, followed by an

abrupt transition to a nonwoody lignin source at 12.3 ka. After 12.3 ka, lignins gradually decreased until they stabilized at ~10.9 ka.

Assessment: The proxy data from the Fallen Leaf Lake sediment cores show some ecosystem changes occurred within the YD chronozone, but the details are uncertain because of the coarse sampling resolution (4 samples that span the entire YD climate event). Although there appears to be at least some evidence for intra-YD climate variability in this part of the Sierra Nevada, unequivocal evidence of a two-stage YD event is not present in this record.

Citation: Ball, G. I., Noble, P. J., Stephens, B. M., Higgins, A., Mensing, S. A., and Aluwihare, L. I., 2018, A lignin, diatom, and pollen record spanning the Pleistocene-Holocene transition at Fallen Leaf Lake, Sierra Nevada, California, USA, *in* Starratt, S. W., and Rosen, M. R., eds., From Saline to Freshwater: The Diversity of Western Lakes in Space and Time: Geological Society of America Special Paper 536, p. 1–18, doi: 10.1130/2018.2536(01).

Lake Barrett and Starkweather Lake, California

Summary: MacDonald et al. (2008) evaluated lacustrine sediments from cores taken at Lake Barrett and Starkweather Lake in the east-central Sierra Nevada, California to examine hydroclimate conditions in the region during the YD climate event. They used radiocarbon dating and measured a number of different hydroclimate proxies, including loss-on-ignition (LOI), chironomid temperature reconstructions, bulk organic $\delta^{13}C$, cellulose-inferred lake water $\delta^{18}O$, diatom salinity and depth reconstructions, planktonic diatom percentages, and pollen percentages, constrained cluster analysis (CONISS) pollen zonation based upon terrestrial pollen, and charcoal. The authors concluded there was an initial increase in effective moisture during the early part of the YD that was followed by drier conditions later in the YD, but did not determine the precise timing of the transition.

Assessment: The proxy data presented (their Figure 4) clearly show variability throughout the YD climate event and there is some evidence of a wet to dry transition during the YD, particularly in the diatom-inferred lake data from Lake Barrett. However, the significance and timing of the transition is difficult to decipher because many of the proxies do not show the same wet-to-dry transition as the diatoms and only one of the seven calibrated ^{14}C ages actually falls within the YD chronozone (their Table 1). Although the data suggest a possible two-staged YD event in this part of the Sierra Nevada, unequivocal evidence of such an event is not present in this record.

Citation: MacDonald, G. M., Moser, K. A., Bloom, A. M., Porinchu, D. F., Potito, A. P., Wolfe, B. B., Edwards, T. W. D., Petel, A., Orme, A. R., and Orme, A. J., 2008, Evidence of temperature depression and hydrological variations in the eastern Sierra Nevada during the Younger Dryas stade: Quaternary Research, v. 70, p. 131-140.

Moaning Cave, California

Summary: Oster et al. (2009) used uranium-series dating and measured stable isotopes ($\delta^{18}O$, $\delta^{13}C$), trace elements, and strontium isotopes in a speleothem from Moaning Cave, California that spans much of the late glacial period. Following an initial decrease in $\delta^{18}O$ and $\delta^{13}C$ and trace element values at 12.4 ka, which they correlate to the beginning of the YD, the isotopic and trace element values increase slightly at ~12.2 ka. $^{87}Sr/^{86}Sr$ values also increase slightly at this time. The authors interpret these changes as suggesting "a decrease in the contribution of soil-respired CO_2 to seepage waters and an increase in prior calcite precipitation coupled with increased water–host-rock interactions, consistent with a slight mid-YD increase in aridity and possibly temperature in this region." An additional shift in isotopic values occurred at ~12.1 ka, which was attributed to movement of the stalagmite drip center.

Assessment: Although the Moaning Cave proxy data exhibit variability within the YD (their Figure 3), the significance and timing of the mid-YD variations is not well defined. Specifically, it is unclear if (1) the change in values observed at ~12.4 truly reflect the beginning of the YD (this is ~500 years after the beginning of the YD event as defined in the Greenland ice cores), (2) the changes at ~12.2 ka are the result of a change in local hydroclimate conditions (the authors do not elaborate on how a slight increase in aridity would affect these proxy systems), and (3) the changes at ~12.1 ka reflect movement of the stalagmite drip center (and not a change in climate) as hypothesized. Moreover, uncertainties in the age dating of the speleothem within the YD timeframe are on the order of ±450 years, which precludes robust comparison with other proxy records. Although the Moaning Cave data indicate unstable conditions likely prevailed in this part of the Sierra Nevada during the YD climate event, they do not provide unequivocal evidence of two distinct stages within the event itself.

Citation: Oster, J. L., Montañez, I. P., Sharp, W. D., and Cooper, K. M., 2009, Late Pleistocene California droughts during deglaciation and Arctic warming: Earth and Planetary Science Letters, v. 288, p. 434-443.

Swamp Lake, California

Summary: Street et al. (2012) investigated sediment cores taken at Swamp Lake in the central Sierra Nevada, California to reconstruct climatic changes on millennial and centennial timescales over the past ~20,000 years. They used radiocarbon dating and measured total organic carbon (TOC), total nitrogen, C/N, $\delta^{13}C_{org}$, $\delta^{15}N$, biogenic silica, magnetic susceptibility, and sediment lithology at high resolution, and found that climate fluctuated between relatively warm/dry intervals with high TOC and cold/wet intervals characterized by low TOC and high detrital input. Their unit SL-7 dates to 13.1-11.7 ka, which correlates temporally with the YD climate event, and consists of several distinct sedimentary units, including a major sand/gravel layer (13.1-12.8 ka), low TOC laminated and massive gyttja (12.8-12.5 ka), high TOC peat (12.5-12.2 ka), and interbedded clay/gyttja/sand (12.1-11.6 ka), suggesting a complex structure for the YD interval at this site. The authors interpret the lacustrine sediments and chemical/biological proxies to indicate depressed lake productivity and cold, wet conditions during the early part of the YD, followed by partial warming and drying after ~12.5 ka.

Assessment: The sediments and proxy data from the Swamp Lake cores (their Figure 6) clearly show tremendous variability within the YD climate event. The peat layer that dates to ~12.5-12.2 ka is particularly interesting as it indicates a wetland, rather than a lake, prevailed at this time, likely as a result of warmer/drier conditions compared to those prior to 12.5 ka. However, the presence of clay/gyttja/sand above the peat layer suggests a return to cold, wet conditions at the end of the YD, analogous to the interpretations of cold, wet conditions based on the sand and gyttja layers just below the peat. Although the data clearly show unstable climate conditions prevailed during the YD event, unequivocal evidence of two distinct stages within the event itself is not present in this record.

Citation: Street, J. H., Anderson, R. S., and Paytan, A., 2012, An organic geochemical record of Sierra Nevada climate since the LGM from Swamp Lake, Yosemite: Quaternary Science Reviews, v. 40, p. 89-106.

Chihuahuaños Bog, New Mexico

Summary: Cisneros-Dozal et al. (2010) reconstructed hydroclimate conditions over the past 15,000 years based on sediments from a core taken from Chihuahuaños Bog in northern New Mexico. Although evidence of the YD climate event as a whole is equivocal in the bog's pollen record (Anderson et al., 2008), the $\delta^{13}C$, $\delta^{15}N$, C/N, organic carbon profiles and radiocarbon ages clearly demarcate the YD,

which was characterized by low terrestrial productivity typical of cold/wet conditions. In addition, high concentrations of algae during the second half of the YD was interpreted as signifying an increase in aquatic productivity.

Assessment: The proxy data from Chihuahuaños Bog (their Figure 3) exhibit variability during the YD climate event, but only the concentrations of algae increased in the latter part of the YD. Moreover, it is unclear exactly when this increase took place as none of the seven ^{14}C ages reported fall within the YD chronozone. Although there appears to be at least some intra-YD climate variability in this part of New Mexico, unequivocal evidence of a two-stage YD event is not present in this record.

Citation: Cisneros-Dozal, L. M., Heikoop, J. M., Fessenden, J., Anderson, R. S., Meyers, P. A., Allen, C. D., Hess, M., Larson, T., Perkins, G., and Rearick, M., 2010, A 15 000-year record of climate change in northern New Mexico, USA, inferred from isotopic and elemental contents of bog sediments: Journal of Quaternary Science, v. 25, p. 1001-1007.

SI 2b

		**Latitude**	**Longitude**	**Elevation**	**Type of**	**Record**	**Age range** (ka)		**Dates within**	
**Site Name**	**State**	(°N)	(°W)	masl	**deposit**	**type**	min	max	**YD chronozone**	**Proxies**
Bonsai Lake<br>*(Wilcox et al. 2020)*	AK	55.28	133.64	107	lacustrine	core	0.7	12.8	4 of 10<br>(radiocarbon)	% organics<br>C/N<br>$\delta^{13}C$<br>grain size<br>magnetic susceptibility<br>pollen
Discovery Pond<br>*(Kaufman et al. 2010)*	AK	60.79	150.84	97	lacustrine	core	8.3	13.3	0 of 11<br>(radiocarbon)	biogenic silica<br>magnetic susceptibility<br>microfossils<br>organic matter (%)<br>pollen
Mount Waskey<br>*(Young et al. 2019)*	AK	59.87	159.22	287	glacial	glacial<br>erratics	10.4	12.8	16 of 19<br>(cosmogenic ^{10}Be)	inboard erratics<br>moraine boulders<br>upvalley erratics
Dove Springs Wash<br>*(Pigati et al. 2019)*	CA	35.40	118.00	858	wetland	outcrop	11.4	12.7	6 of 7<br>(radiocarbon)	grain size<br>redox properties<br>organic content<br>microfauna (gastropods)
Fallen Leaf Lake<br>*(Ball et al. 2018)*	CA	38.90	120.06	1942	lacustrine	core	9.0	13.5	2 of 14<br>(radiocarbon)	$\delta^{13}C$<br>diatoms<br>lignin phenol<br>magnetic susceptibility<br>pollen<br>total organic content
Lake Barrett<br>*(MacDonald et al. 2008)*	CA	37.60	119.01	2816	lacustrine	core	8.6	13.8	0 of 4<br>(radiocarbon)	charcoal<br>Cheno/Am pollen<br>chironomid temperature<br>diatom lake depth<br>diatom salinity<br>LOI<br>planktonic diatoms<br>pollen zonation
Little Dixie Wash<br>*(Pigati et al. 2019)*	CA	35.56	117.85	836	wetland	outcrop	11.1	12.6	2 of 4<br>(radiocarbon)	grain size<br>redox properties<br>organic content<br>microfauna (gastropods)
Mesquite Springs<br>*(Pigati et al. 2019)*	CA	35.01	116.21	309	wetland	outcrop	12.4	12.7	4 of 4<br>(radiocarbon)	grain size<br>redox properties<br>organic content<br>microfauna (gastropods)
Moaning Cave<br>*(Oster et al. 2009)*	CA	--	--	--	speleothem	core	8.8	16.9	2 of 14<br>(U-series)	$^{87}Sr/^{86}Sr$<br>$\delta^{13}C$<br>$\delta^{18}O$<br>geochemistry - trace elements
Starkweather Lake<br>*(MacDonald et al. 2008)*	CA	37.66	119.07	2424	lacustrine	core	10.6	13.3	1 of 3<br>(radiocarbon)	bulk organic $\delta^{13}C$<br>cellulose-inferred lake water $\delta^{18}O$<br>charcoal<br>Cheno/Am pollen<br>chironomid temperature<br>LOI<br>planktonic diatoms<br>pollen zonation
Swamp Lake<br>*(Street et al. 2012)*	CA	37.95	119.82	1554	lacustrine	core	0.6	18.0	1 of 13<br>(radiocarbon)	biogenic silica<br>C/N<br>$\delta^{13}C_{org}$<br>$\delta^{15}N$<br>magnetic susceptibility<br>total nitrogen<br>total organic content
Chihuahueños Bog<br>*(Cisneros-Dozal et al. 2010)*	NM	36.05	106.51	2925	wetland	core	0.0	13.7	0 of 7<br>(radiocarbon)	C/N<br>$\delta^{13}C$<br>$\delta^{15}N$<br>extractable Pb<br>organic carbon (%)<br>pollen

SI 3a

Laboratory methods

We used radiocarbon (^{14}C) dating of charcoal, organic material, and small terrestrial gastropod shells of the Succineidae family to establish age control for the DEVA paleospring deposits. Charcoal and organic material are recognized as suitable material for ^{14}C dating paleowetland deposits [1-6]. Previous work has shown the Succineidae shells also yield reliable ^{14}C ages regardless of the local habitat, environmental conditions, rock type, and geologic setting [7, 8], including desert wetland ecosystems in the southwestern U.S. [3-5, 9].

Charcoal and organic matter samples were treated at the U.S. Geological Survey's Radiocarbon Laboratory in Denver, CO using either the standard acid-base-acid (ABA) procedure or a modified procedure that involves using HF to concentrate the organic material prior to the ABA treatment (HF/ABA). The ABA treatment consisted of an initial acid wash with 1N HCl at 60 °C for 30 minutes to remove carbonate minerals, a base wash with 1N NaOH at 60 °C for 30 minutes to remove humic acids and other base-soluble compounds (this step was repeated until the supernatant remained clear), and a final acid wash in 1N HCl at 60 °C for at least 10 minutes to acidify the sample. The HF/ABA treatment included an initial acid wash with 1N HCl at 60 °C for 30 minutes to remove carbonate minerals, immersion of the sample in concentrated HF at room temperature for at least 30 minutes to remove silicate minerals, and a second wash in 1N HCl at 60 °C for at least 10 minutes to prevent precipitation of fluoride minerals. These steps were followed by the base and final acid treatments of the standard ABA procedure. Following both procedures, the treated samples were washed with ASTM Type 1, 18.2 MΩ water and dried.

Clean, dry gastropod shells selected for dating were broken and examined under a dissecting microscope to ensure that the interior whorls were free of secondary carbonate and detritus. Fossil shells that were free of detritus were bleached with 30% H_2O_2 to remove organic matter and etched with dilute HCl to remove 30-50% of the total mass prior to hydrolysis (H_2O_2/HCl). We selected several shells for X-ray diffraction analysis to verify that only shell aragonite remained prior to preparation for ^{14}C analysis. None of the shells that we analyzed contained measurable quantities of calcite.

Pretreated charcoal and organic matter samples were initially heated to 150 °C for 15 minutes to drive off atmospheric gases and then combusted online at 625 °C in the presence of excess high-purity oxygen. Shell carbonate was converted to CO_2 using American Chemical Society (ACS) reagent grade 85% H_3PO_4 under vacuum at 80 ºC until the reaction was visibly complete (~1 hr). For all samples, water and other contaminant gases were removed from the sample CO_2 by precise cryogenic separation at -140 °C using a variable temperature trap capable of holding temperatures to within 1-2 °C of the desired target. The resulting purified CO_2 gas was measured manometrically, converted to graphite using an iron catalyst and the standard hydrogen reduction process [10], and submitted for AMS ^{14}C analysis.

All ^{14}C ages were calibrated using the IntCal20 dataset and CALIB 8.2html [11, 12]. Ages are presented in thousands of calibrated years before present (ka = thousands of years before present; 0 ka = 1950 CE), and uncertainties are given at the 95% (2σ) confidence level.

Age modeling

We used the calibrated ages to model the kernel density estimate (KDE) distribution of the Death Valley paleospring deposits. KDE modeling is a hybrid Bayesian/frequentist approach that is used to determine and graphically represent the underlying distributions of discrete data points [13]. In this study, KDE modeling was done using the `KDE_Model` function in OxCal 4.4.2 which employs a Markov Chain Monte Carlo (MCMC) implementation to generate an equal number of random samples from each of the events specified within the kernel probability distribution. We used the default values in OxCal for both the kernel and bandwidth estimates to evaluate the age distribution. During this process, the model generated snapshots of the KDE distribution at increments of 1000 iterations, as well as the summed probability distribution of the ages for reference (Fig. 3). In addition, the starting age of the YD, the mid-YD transition, and the end of the YD were determined using the `Boundary` function in OxCal (Fig. 3).

References

1. Quade J, Mifflin MD, Pratt WL, McCoy WD, Burckle L. Fossil spring deposits in the southern Great Basin and their implications for changes in water-table levels near Yucca Mountain, Nevada, during Quaternary time. *Geological Society of America Bulletin* 1995, **107**(2)**:** 213-230.

2. Quade J, Forester RM, Pratt WL, Carter C. Black mats, spring-fed streams, and late-glacial-age recharge in the southern Great Basin. *Quaternary Research* 1998, **49:** 129-148.

3. Pigati JS, Miller DM, Bright JE, Mahan SA, Nekola JC, Paces JB. Chronology, sedimentology, and microfauna of groundwater discharge deposits in the central Mojave Desert, Valley Wells, California. *Geological Society of America Bulletin* 2011, **123**(11/12)**:** 2224-2239.

4. Springer KB, Manker CR, Pigati JS. Dynamic response of desert wetlands to abrupt climate change. *Proceedings of the National Academy of Sciences USA* 2015, **112**(47)**:** 14522-14526.

5. Springer KB, Pigati JS, Manker CR, Mahan SA. The Las Vegas Formation. *US Geological Survey Professional Paper 1839, 62 p* 2018**:** doi: 10.3133/pp1839.

6. Springer KB, Pigati JS. Climatically driven displacement on the Eglington fault, Las Vegas, Nevada, USA. *Geology* 2020, **48**(6)**:** 574-578.

7. Pigati JS, Rech JA, Nekola JC. Radiocarbon dating of small terrestrial gastropod shells in North America. *Quaternary Geochronology* 2010, **5:** 519-532.

8. Pigati JS, McGeehin JP, Muhs DR, Bettis EAI. Radiocarbon dating late Quaternary loess deposits using small terrestrial gastropod shells. *Quaternary Science Reviews* 2013, **76:** 114-128.

9. Pigati JS, Bright JE, Shanahan TM, Mahan SA. Late Pleistocene paleohydrology near the boundary of the Sonoran and Chihuahuan Deserts, southeastern Arizona, USA. *Quaternary Science Reviews* 2009, **28:** 286-300.

10. Vogel JS, Southon JR, Nelson DE, Brown TA. Performance of catalytically condensed carbon for use in accelerator mass spectrometry. *Nuclear Instruments and Methods in Physics Research B* 1984, **5:** 289-293.

11. Reimer PJ, Austin WEN, Bard E, Bayliss A, Blackwell PG, BronkRamsey C*, et al.* The IntCal20 Northern Hemisphere radiocarbon age calibration curve (0-55 cal kBP). *Radiocarbon* 2020, **62:** 725-757.

12. Stuiver M, Reimer PJ, Reimer RW. *CALIB 82 [WWW program] at http://caliborg, accessed 2020-8-23* 2020.

13. Bronk Ramsey C. Methods for summarizing radiocarbon datasets. *Radiocarbon* 2017, **59**(6)**:** 1809-1833.

SI 3b

Sample #	AMS #	Depth (cm)[1]	Context	Material dated	Treatment[2]	^{14}C age (ka)	Cal age (ka)[3]	Intercept 1 (ka)[4]	P[5]	Intercept 2 (ka)[4]	P[5]	Intercept 3 (ka)[4]	P[5]	Intercept 4 (ka)[4]	P[5]
Section 18DEVA4-30.2															
18DEVA4-30.2b	USGS-1064	5	sediment	charcoal	ABA	10.16 ± 0.05	**11.79 ± 0.18**	11.61 - 11.97	0.93						
18DEVA4-30.2e	USGS-1065	62	sediment	charcoal	ABA	10.12 ± 0.05	**11.74 ± 0.14**	11.60 - 11.88	0.74	11.47 - 11.59	0.14	11.40 - 11.45	0.06		
18DEVA4-30.2d	USGS-1076	75	sediment	Succineidae	H_2O_2/HCl	10.20 ± 0.04	**11.87 ± 0.13**	11.74 - 12.00	0.95						
18DEVA4-30.2f	USGS-1062	115	black mat	organic matter	ABA	10.71 ± 0.05	**12.71 ± 0.05**	12.66 - 12.76	0.90	12.62 - 12.65	0.06				
18DEVA4-30.2g	USGS-1077	155	sediment	Succineidae	H_2O_2/HCl	10.83 ± 0.05	**12.78 ± 0.06**	12.72 - 12.84	0.95						
18DEVA4-30.2k	USGS-1773	170	sediment	Succineidae	H_2O_2/HCl	10.99 ± 0.05	**12.89 ± 0.12**	13.02 - 13.07	0.10	12.77 - 13.01	0.86				
18DEVA4-30.2h	USGS-1078	185	sediment	Succineidae	H_2O_2/HCl	11.11 ± 0.06	**13.01 ± 0.12**	12.89 - 13.12	0.92						
Section 19DEVA11-11.1															
19DEVA11-11.1k	USGS-1741	3	sediment	charcoal	HF/ABA	10.17 ± 0.04	**11.82 ± 0.13**	11.69 - 11.95	0.91						
19DEVA11-11.1l	USGS-1742	15	black mat	organic matter	HF/ABA	10.26 ± 0.05	**12.00 ± 0.18**	12.39 - 12.45	0.06	11.81 - 12.18	0.84				
19DEVA11-11.1n	USGS-1743	31	sediment	charcoal	HF/ABA	10.27 ± 0.04	**11.96 ± 0.14**	11.82 - 12.11	0.86						
19DEVA11-11.1o	USGS-1744	31	sediment	charcoal	HF/ABA	10.34 ± 0.04	**12.08 ± 0.13**	12.35 - 12.47	0.25	12.30 - 12.33	0.07	12.22 - 12.28	0.10	11.95 - 12.20	0.54
19DEVA11-11.1p	USGS-1745	38	sediment	charcoal	HF/ABA	10.34 ± 0.04	**12.08 ± 0.13**	12.35 - 12.47	0.25	12.30 - 12.33	0.07	12.22 - 12.28	0.10	11.95 - 12.20	0.54
19DEVA11-11.1q	USGS-1746	49	black mat	organic matter	HF/ABA	10.42 ± 0.04	**12.30 ± 0.20**	12.54 - 12.60	0.08	12.10 - 12.49	0.86				
19DEVA11-11.1t	USGS-1947	241	sediment	Succineidae	H_2O_2/HCl	10.89 ± 0.05	**12.79 ± 0.05**	12.74 - 12.85	0.87	12.86 - 12.89	0.09				

Uncertainties for the calibrated ages are given at the 2σ (95%) confidence level. All other uncertainties are given at 1σ (68%).

[1] Depth from the top of the YD-age unit within each stratigraphic section.

[2] ABA = acid-base-acid; HF/ABA = HF treatment followed by ABA; H_2O_2/HCl = bleach followed by acid leach (see SI 3a for details).

[3] Calibrated ages were calculated using OxCal v.4.4.2, IntCal20.14C dataset; limit 55.0 calendar ka B.P. and are reported as the midpoint of the intercept range with the highest probability. Uncertainties are calculated as the difference between the midpoint and either the upper or lower limit of that calibrated age range, whichever is greater.

[4] Age ranges are reported when the probability of a calibrated age range exceeds 0.05.

[5] P = probability of the calibrated age falling within the reported range as calculated by OxCal v.4.4.2.

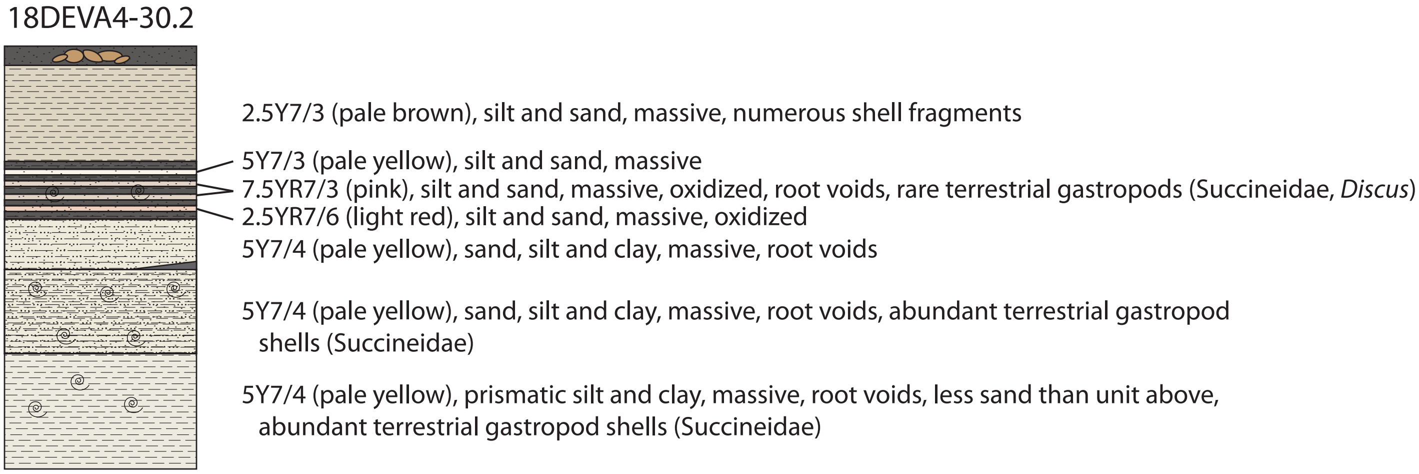

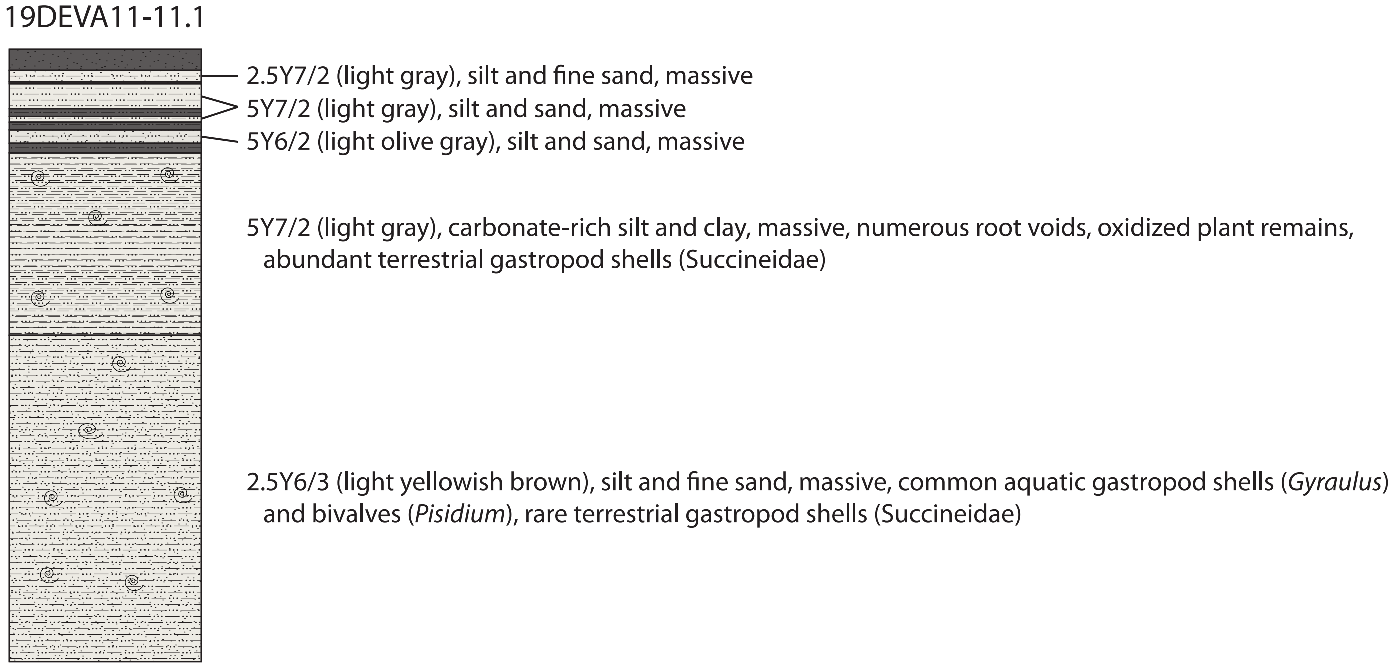

SI 4. Stratigraphic sections with sediment descriptions. Black mats all consist of organic-rich silt and clay. Refer to Figures 1 and 2 for sample locations, calibrated ages, and hydrologic interpretations.
